# Blood vessels in a dish: the evolution, challenges, and potential of vascularized tissues and organoids

**DOI:** 10.3389/fcvm.2024.1336910

**Published:** 2024-06-13

**Authors:** Peter N. Nwokoye, Oscar J. Abilez

**Affiliations:** ^1^ Department of Medicine, Stanford University School of Medicine, Stanford, CA, United States; ^2^ Department of Cardiothoracic Surgery, Stanford University, Stanford, CA, United States; ^3^ Division of Pediatric CT Surgery, Stanford University, Stanford, CA, United States; ^4^ Cardiovascular Institute, Stanford University, Stanford, CA, United States; ^5^ Maternal and Child Health Research Institute, Stanford University, Stanford, CA, United States; ^6^ Bio-X Program, Stanford University, Stanford, CA, United States

**Keywords:** vascularized tissues, pluripotent stem cells, 3D bioprinting, microfluidics, bioinks, vascular pathology, microvessels

## Abstract

Vascular pathologies are prevalent in a broad spectrum of diseases, necessitating a deeper understanding of vascular biology, particularly in overcoming the oxygen and nutrient diffusion limit in tissue constructs. The evolution of vascularized tissues signifies a convergence of multiple scientific disciplines, encompassing the differentiation of human pluripotent stem cells (hPSCs) into vascular cells, the development of advanced three-dimensional (3D) bioprinting techniques, and the refinement of bioinks. These technologies are instrumental in creating intricate vascular networks essential for tissue viability, especially in thick, complex constructs. This review provides broad perspectives on the past, current state, and advancements in key areas, including the differentiation of hPSCs into specific vascular lineages, the potential and challenges of 3D bioprinting methods, and the role of innovative bioinks mimicking the native extracellular matrix. We also explore the integration of biophysical cues in vascularized tissues *in vitro*, highlighting their importance in stimulating vessel maturation and functionality. In this review, we aim to synthesize these diverse yet interconnected domains, offering a broad, multidisciplinary perspective on tissue vascularization. Advancements in this field will help address the global organ shortage and transform patient care.

## 1 Introduction

Vascular pathologies are a prevalent and often critical aspect of human health, influencing a broad spectrum of diseases (1–3). The ubiquity of these conditions necessitates a deeper understanding of vascular biology, which has been significantly advanced by the development of *in vitro* models. The global organ shortage, a crisis exacerbated by an aging population and a growing prevalence of chronic diseases, underscores the urgent need for innovative solutions (4–6). Within this context, the potential of vascularized tissues, engineered *in vitro*, offers a promising avenue to address the escalating demand for transplantable organs.

Vascularization is critical in overcoming the oxygen and nutrient diffusion limit of approximately 100–200 μm, a threshold beyond which tissue viability drastically decreases (7). Establishing a perfusable vascular network within engineered tissues is essential for replicating the dynamic microenvironment of native tissues, facilitating the removal of metabolic waste, and maintaining cellular homeostasis. This is especially crucial for the development of thick, complex tissue constructs. Advanced vascularization techniques enhance the translational potential of tissue models in drug discovery, offering a more accurate representation of drug pharmacokinetics and pharmacodynamics. In regenerative medicine, vascularized grafts ensure immediate integration and long-term survival post-implantation (8–10).

The evolution of vascularized tissues in a dish represents a convergence of multiple scientific disciplines, marked by significant progress and challenges. A critical component of this evolution is the differentiation of human pluripotent stem cells (hPSCs) into vascular cells (11–13). The ability to guide hPSCs towards specific vascular lineages has been a focus of intense research, offering insights into developmental biology, while paving the way for creating functional vascular networks. There is still an unmet need for constructing larger vessels *in vitro* without relying on animal hosts (14).

Advancements in three-dimensional (3D) bioprinting have been instrumental in tissue engineering, particularly for tissue vascularization (15, 16). Techniques such as extrusion-based bioprinting, along with innovative approaches like sacrificial writing into functional tissue (SWIFT) and freeform reversible embedding of suspended hydrogels (FRESH), have shown remarkable potential in creating complex, vascularized tissue structures. These methods allow for the precise deposition of cells and biomaterials, enabling the fabrication of tissues with intricate vascular networks. However, the field of 3D bioprinting still grapples with challenges, including the need for high-resolution printing capabilities and maintaining cell viability and function post-printing (17, 18).

The development and improvement of bioinks, particularly smart hydrogels, have been critical in advancing 3D bioprinting technologies (19). These materials provide the necessary support for cell growth and differentiation and mimic the native extracellular matrix (ECM). Moreover, integrating responsive materials that can adapt to environmental stimuli further enhances the potential of these bioinks, creating dynamic, lifelike tissues (20).

**Figure 1:**
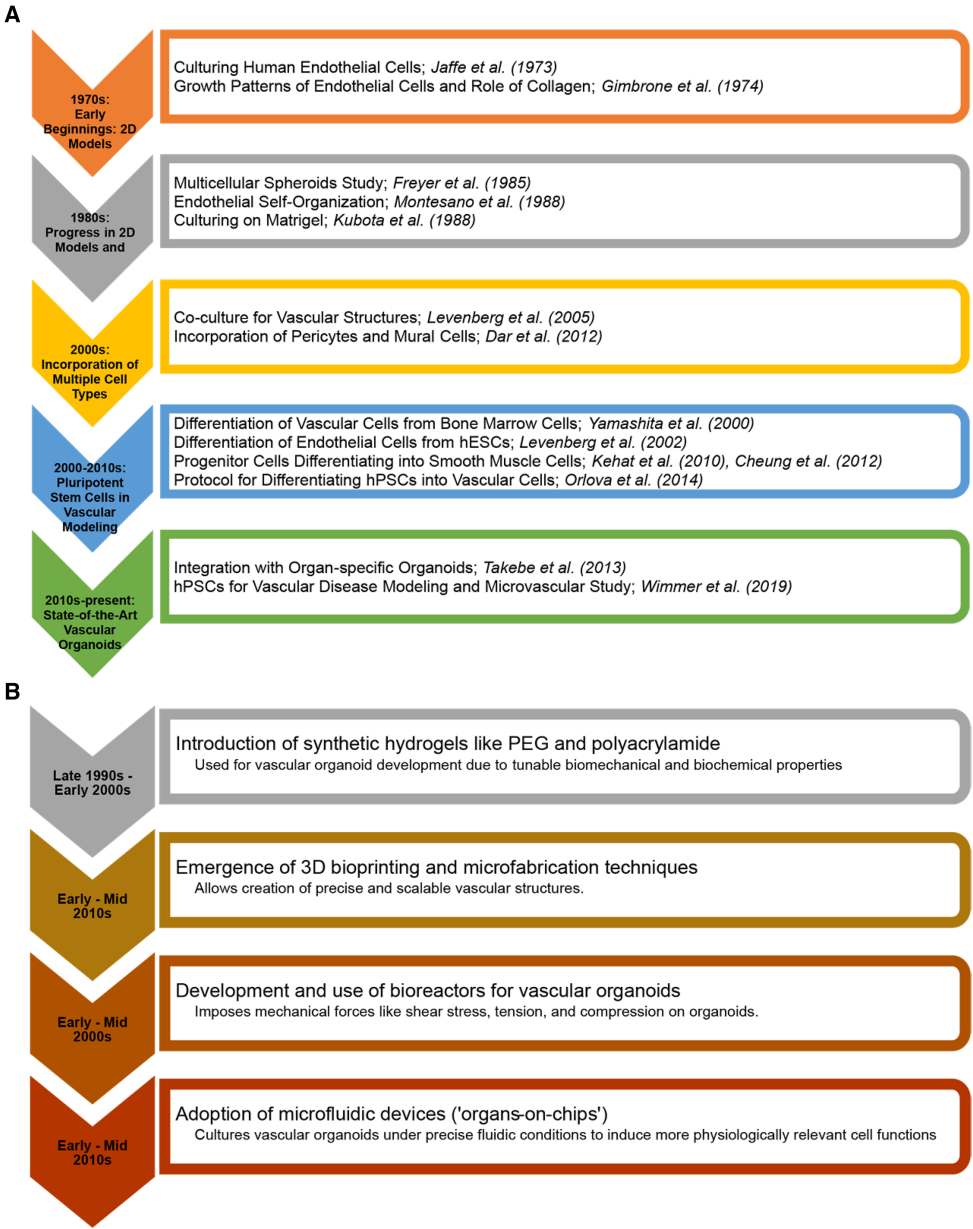
Milestones in vascular biology and tissue engineering in the evolution of vascular tissues and organoids. (**A**) From the early cultivation of endothelial cells to the advanced integration of pluripotent stem cell-derived vascular structures with (**B**) 3D bioprinting and organ-on-chip technologies. The charts highlight key breakthroughs and the progressive sophistication of *in vitro* vascular tiissues and organoids for regenerative medicine and disease modelling.

Microfluidics has emerged as a vital tool in the *in vitro* study of vascularized tissues (21, 22). The ability to perfuse these tissues in a controlled manner is essential for their survival, maturation, and functionality (23). Microfluidic systems enable the simulation of physiological blood flow, providing insights into vascular biology and pathology under conditions that closely resemble the *in vivo* environment (24). This technology is crucial for understanding the mechanical and biochemical cues that influence vascular development and disease progression. One of the overarching goals of vascular tissue engineering is to reduce reliance on animal models (25). By creating human-relevant, vascularized tissues *in vitro*, it is possible to bypass the myriad ethical and scientific limitations associated with animal hosts. This will accelerate the path to clinical translation and improve the relevance and accuracy of preclinical models. Figure 1 shows milestones in vascular biology and tissue engineering in the evolution of vascular tissues and organoids.

Considering the ideas above, this review aims to analyze the current state and future potential of vascularized tissues and organoids engineered *in vitro*. We begin with a discussion of microvessels, then uniquely synthesize the past with recent advancements in hPSC differentiation, 3D bioprinting technologies like SWIFT and FRESH, improvements in bioinks, and the integration of biophysical factors for tissue perfusion. By consolidating these diverse yet interconnected domains, this review adds a broad multidimensional perspective on tissue and organoid vascularization.

## 2 Microvessels in vascular biology and pathology

**Figure 2:**
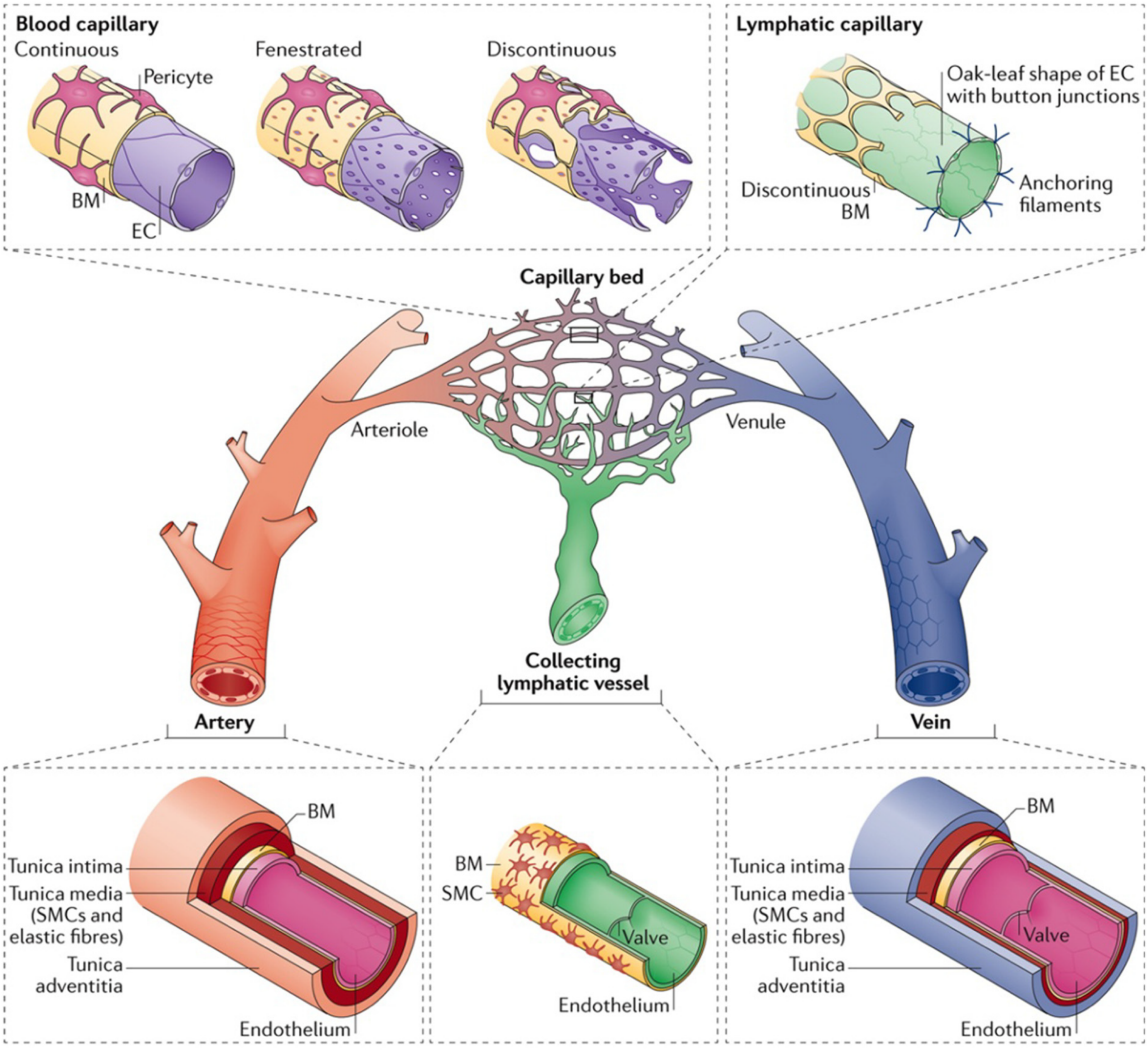
The hierarchy and complexities of human vascular and lymphatic networks. This figure delineates the structural distinctions and functional nuances between arteries, arterioles, capillary beds, venules, veins, and lymphatic vessels. Arteries have robust walls comprising smooth muscle and elastic fibers. They taper into arterioles, which regulate blood flow into capillary networks. Capillaries are shown in three morphologies: *continuous,* with unbroken endothelial linings for restricted permeability; *fenestrated,* with pores to facilitate exchange; and *discontinuous,* allowing greater movement of cells and molecules. Venules collect blood from capillaries, leading to veins, which are equipped with valves to direct venous blood return. Lymphatic capillaries are uniquely structured with button-like junctions for fluid entry, converging into collecting vessels containing valves. Used with permission from Potente et al. (27) Copyright © 2017, Springer Nature Limited.

Microvessels are the smallest components of the circulatory system and are widely studied for their roles in vascular biology and pathology. These microscopic vessels, comprising arterioles, capillaries, and venules, are vital for tissue perfusion, facilitating efficient gas exchange, nutrient delivery, and waste removal at the cellular level (Figure 2) (26, 27). Understanding their roles in physiological and pathological processes requires exploring each type of microvessel. This section briefly touches on the distinct structural features, functional mechanisms, and hemodynamic properties of each microvessel.

### 2.1 Arterioles

Arterioles are the smallest branches of arteries with a diameter ranging from 10 to 100 micrometers. Their structure varies slightly based on location and the specific tissues they supply. These vessels are surrounded by smooth muscle cells, enabling precise blood flow regulation. Histologically, arterioles comprise three concentric layers: the tunica intima (innermost), tunica media, and tunica externa (outermost) (28). The tunica intima comprises a single layer of endothelial cells on a basement membrane. These endothelial cells regulate vascular tone and permeability and mediate inflammatory and coagulation processes. The tunica media comprises smooth muscle cells in a circular arrangement and regulates blood flow by facilitating arteriolar vasoconstriction or vasodilation (29). The tunica externa, composed of connective tissues, provides structural support, and anchors the microvessel to surrounding tissues. Blood flow regulation in arterioles is a dynamic process influenced by hemodynamic forces such as shear stress (30). This process primarily involves vasoconstriction and vasodilation in response to physiological stimuli, including neural, hormonal, and local metabolites. The nitric oxide production by endothelial cells plays a significant role in arteriolar vasodilation, directly impacting tissue perfusion and health (31).

Arteriolar dysfunction is implicated in a variety of vascular pathologies. Hypertension arises from increased arteriolar resistance due to arteriolar constriction or structural changes like arteriosclerosis (32). Arteriolar constriction or obstruction can precipitate ischemic stroke by diminishing blood flow, thus inducing brain tissue damage. Conversely, arteriolar weakening can lead to hemorrhagic stroke. Peripheral artery disease manifests as a consequence of arteriolar dysfunction, resulting in insufficient blood supply to the limbs, which can cause pain, cramps, and tissue necrosis in extreme cases (33). Arteriolar damage induces diabetic microvascular complications, such as retinopathy, nephropathy, and neuropathy, primarily through impaired blood flow and increased vascular permeability (34). In diabetic or hypertensive retinopathy, arteriolar abnormalities contribute to retinal damage. Furthermore, arteriolar dysfunction can cause preeclampsia, characterized by high blood pressure (35). Finally, Raynaud's phenomenon involves transient arteriolar vasoconstriction, reducing blood flow to extremities and causing numbness and cold sensations in response to cold temperatures or stress (36).

### 2.2 Capillaries

Capillaries have a diameter ranging from 5 to 10 μm and consist of a single endothelial cell layer and a basement membrane (37). They are thus the key sites for fluid and solute exchange between blood and tissues. Hemodynamically, capillaries feature low-velocity flow with a high surface area, which optimizes gas, nutrient, and waste exchange. Capillaries are remarkably diverse in structure and function, reflecting the local microenvironment and tissue demands (38). Histologically, capillaries can be continuous, fenestrated, or sinusoidal. These capillary types have distinct molecular profiles, determining their permeability and molecular transport selectivity. Continuous capillaries have unbroken endothelial cells forming a complete barrier. They are prevalent in muscles, skin, and the central nervous system (CNS). Tight junction proteins, particularly claudin and occludin, are essential in these capillaries, maintaining the blood-brain barrier and regulating molecule passage in the CNS (39). Fenestrated capillaries have pores in their endothelium, which facilitate the passage of larger molecules. These capillaries are found in organs like kidneys, intestines, and endocrine glands. The fenestrations in these capillaries are covered by a glycocalyx diaphragm, which aids filtration and secretion (40). Finally, sinusoidal capillaries, or sinusoids, have the largest fenestrations and are thus the most permeable. Sinusoids are found in the liver, spleen, and bone marrow, allowing the passage of more significant substances.

The diverse structures and functions of capillaries underlie their essential role in several physiological and pathological processes. Capillary dysfunction has been implicated in several vascular pathologies. Coronary heart disease is influenced by inadequate capillary density or dysfunctional capillary growth, resulting in myocardial ischemia (41). Similarly, heart failure is marked by capillary rarefaction, which diminishes oxygen and nutrient delivery to cardiac tissues (42). In the context of neurological disorders, capillary dysfunction is a critical factor in ischemic stroke, as it disrupts cerebral blood flow and worsens brain tissue damage (43).

Moreover, cerebral capillary dysfunction, which impairs blood-brain barrier integrity, is increasingly recognized as a contributor to the pathogenesis of Alzheimer's disease (AD) (44). In addition to cardiovascular and neurological diseases, capillary dysfunctions underpin respiratory diseases. Chronic obstructive pulmonary disease (COPD) is characterized by reduced capillary density, impacting gas exchange and resulting in hypoxemia (45). Pulmonary hypertension involves dysfunctional lung capillary growth and remodeling (2). In cancer, tumor growth is closely associated with new vessel formation (angiogenesis). The new capillaries formed are structurally and functionally aberrant, facilitating tumor growth and metastasis. Autoimmune diseases also demonstrate capillary involvement. In rheumatoid arthritis, increased capillary permeability is observed in inflamed synovium, contributing to joint damage (46). Systemic sclerosis is characterized by capillary damage and loss, causing tissue ischemia and fibrosis (47).

### 2.3 Venules

Venules are microvessels that function as intermediaries between capillaries and veins and are essential for the return of deoxygenated blood from the tissues to the heart. Histologically, venules comprise three primary layers: endothelium, basement membrane, and pericytes. The endothelium regulates exchange and interacts with blood components; the basement membrane provides structural stability and a regulatory interface; and the pericytes support vascular integrity and regulate blood flow. Venules express various molecular markers, such as PECAM-1, VE-cadherin, ICAM-1, and VEGFR, that are crucial for their functions and identification (48). Compared to arterioles, the hemodynamics within venules are characterized by low pressure and velocity. The low flow rate in venules facilitates the migration of white blood cells into tissues.

The structure of venules varies slightly based on their size and location. There are two primary types of venules: post-capillary venules and muscular venules. Post-capillary venules are the smallest venules, typically measuring between 10 and 30 micrometers in diameter, and are characterized by very sparse or absent layers of smooth muscle cells (49). Their endothelial cells are particularly permeable, facilitating the efficient exchange of gases, nutrients, and metabolic wastes between the blood and surrounding tissues. Muscular venules are larger than post-capillary venules, with diameters ranging from 30 to 100 μm (50). These capillaries are found downstream of post-capillary venules and feature more layers of smooth muscle cells as they increase in size. The smooth muscles enable these venules to respond to various stimuli, such as neural input or circulating hormones, adjusting their diameters to regulate blood pressure and flow (51).

Venular dysfunction plays a significant role in various vascular pathologies, including inflammatory diseases, cancer metastasis, and neurovascular disorders. Chronic inflammation, as seen in rheumatoid arthritis, is associated with a pathological increase in venular permeability (52). The increased permeability results in leukocyte extravasation from the venules to joint tissues, contributing to swelling, pain, and destruction of joint structures. Moreover, systemic lupus erythematosus involves venular inflammation and dysfunction (53). Venous thrombosis arises from venular damage, contributing to blood clot formation within veins (54). Heart failure, liver cirrhosis, and certain kidney diseases are associated with edema, which results from fluid leakage into tissues due to increased venular permeability (55, 56). Dysfunction in venular walls can facilitate cancer metastasis as tumor cells invade venules to enter the circulation and metastasize. In conditions like chronic venous insufficiency, the dysfunction of venules impacts blood return from the limbs to the heart, resulting in edema and skin changes (57, 58). Multiple sclerosis is associated with alterations in venular walls, which contribute to disrupting the blood-brain barrier and subsequent neuronal damage (59, 60). Venular dysfunction, including increased permeability and impaired blood flow regulation, is involved in the onset of septic shock and multi-organ failure (61, 62).

## 3 Evolution of vascularized tissues and organoids

### 3.1 Early beginnings

**Figure 3:**
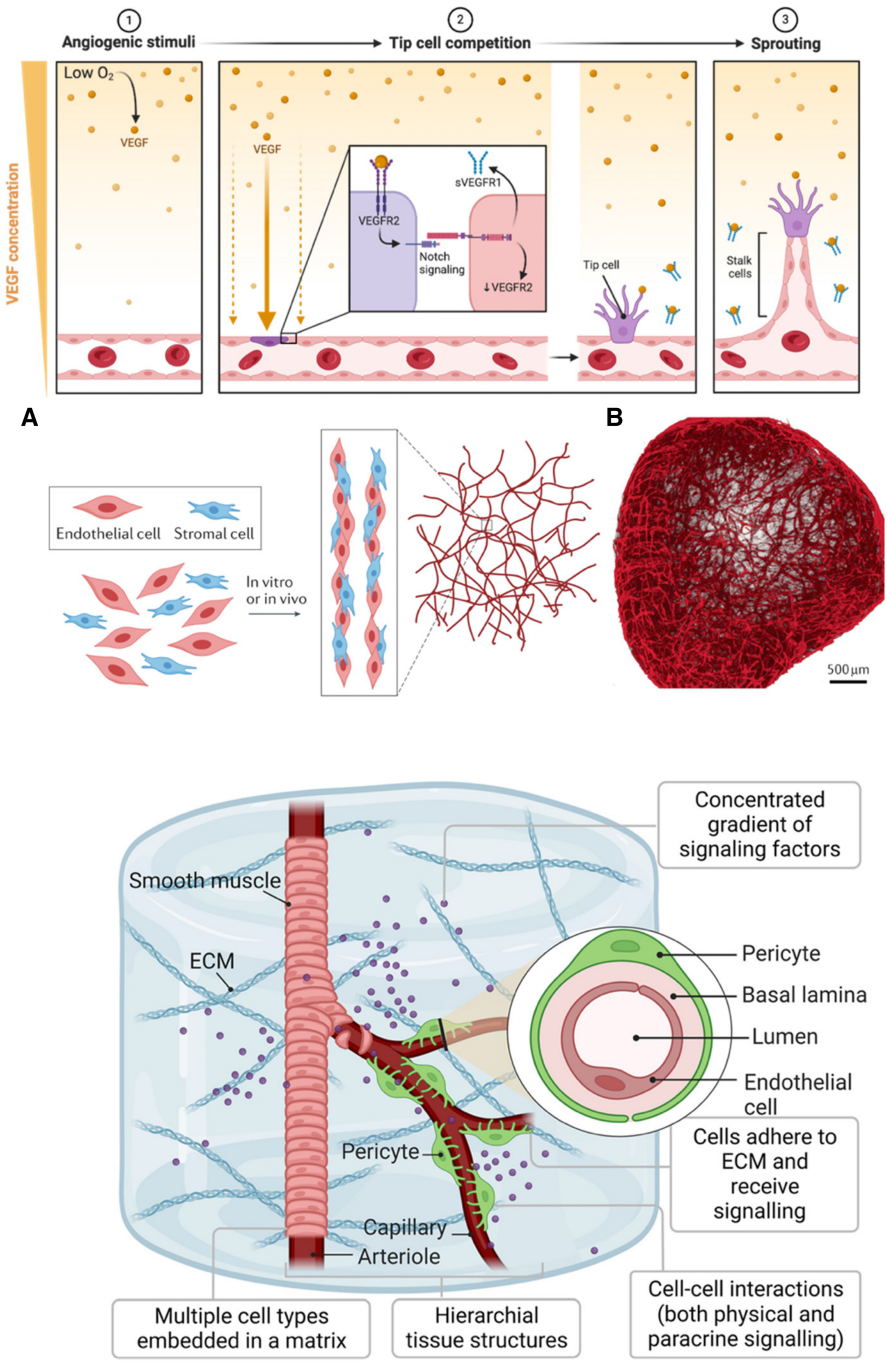
Generating vasculature. (**Top**): A gradient of VEGF initiates angiogenesis in response to hypoxia (1). When oxygen levels are low, VEGF, a protein cells produce, diffuses away from its source, creating a concentration gradient. Endothelial cells towards the lower oxygen gradient respond by expressing VEGF receptors like VEGFR, which, upon binding to VEGF, activate signaling pathways, including Notch (2). The activation of these pathways leads to the selection of tip cells that migrate toward the VEGF source. Soluble VEGFR1 (sVEGFR1) sequesters VEGF to fine-tune this gradient. Tip cells, once established, exhibit filopodia with VEGFR2 to navigate the gradient, while trailing stalk cells elongate the nascent vessels (3). This orchestrated sequence results in the sprouting of new blood vessels. The figure was created with BioRender. (**Middle**): (**A**) Assembly and maturation process of vascular structures, showing the interaction between endothelial cells (red) and stromal cells (blue). These cells undergo co-culture either *in vitro* or *in vivo,* leading to the formation of capillary-like networks. (**B**) Blood vessel organoid formed from self-assembly of endothelial cells. Used with permission from O'Connor et al. (63) Copyright © 2022, Springer Nature Limited and Wimmer et al. (64) Copyright © 2019, Springer Nature Limited. (**Bottom**): Development of iPSC-derived vessel within a 3D ECM. The vessels exhibit hierarchy, with a central arteriole that branches into capillaries, flanked by smooth muscle and pericyte cells. The arteriole's smooth muscle transitions to pericytes as it bifurcates into thinner capillaries. The ECM serves as a scaffold where multiple cell types are embedded. The inset is a vessel cross-section showing the concentric layers of the pericyte, basal lamina, and lumen, surrounded by endothelial cells. Used with permission from Naderi-Meshkin et al. (65) licensed under CC BY 4.0.

Endothelial cells line the intimal surfaces of blood and lymphatic vessels and are essential in vascular biology. They are highly specialized cells mediating vasculogenesis, new blood vessel formation from mesenchymal precursors, and angiogenesis, developing new blood vessels from existing ones (Figure 3; Top). These functions make endothelial cells one of the primary cells to generate vascularized tissues. The last decade has witnessed the generation of vascularized organoid models of the brain (66–70), kidney (71), blood vessels (64, 72), liver (73), and heart (74). However, earlier efforts at generating vessel structures primarily involved endothelial cell monocultures. These initial studies provided crucial insights into endothelial cell behavior, growth patterns, and functional properties *in vitro*. We examine vital findings from the early 1970s and onwards that paved the way for current vascularized tissue models.

Jaffe et al. successfully cultured human umbilical vein endothelial cells (HUVECs) using morphologic and immunologic factors to validate the endothelial lineage of the cultured cells (75). Since then, HUVECs have become a cornerstone in tissue engineering and vascular research due to their ease of isolation and robust growth characteristics. These cells are commonly isolated from the umbilical vein using enzymatic methods, typically collagenase, which preserves their viability and functionality (76). HUVECs have served as a model for studying physiological and pathological processes, including angiogenesis (77), atherosclerosis (78), and chronic inflammatory diseases (79). HUVECs have also been essential for creating vascularized constructs (80) and the endothelialization of synthetic grafts (81). Generally, when culturing endothelial cells, growth factors are added to the culture medium to create an environment conducive to cell growth and differentiation.

Fibroblast growth factor (FGF) was one of the earliest growth factors found to promote endothelial cell culture. Gospodarowicz et al. discovered that bovine vascular endothelial cells require FGF in the culture medium to survive and grow, especially when seeded at low densities (82). Other key growth factors, including vascular endothelial growth factor (VEGF) (83), epidermal growth factor (EGF) (84), insulin-like growth factor (IGF) (85), and platelet-derived growth factor (PDGF) (86), have been discovered and are routinely used in endothelial cell culture. VEGF is the most critical growth factor, which stimulates the formation of new blood vessels and enhances the proliferation and migration of endothelial cells. The specific combination and concentration of growth factors can vary depending on the type of endothelial cells being cultured.

When cultured in optimal conditions, endothelial cells naturally self-organize into complex tubular structures (Figure 3; Middle) (63). Montesano et al. seeded primary endothelial cells as monolayers in a collagen matrix and observed that the cells organized into tube-like structures, mimicking capillary formation (87). This pivotal finding demonstrated the inherent ability of endothelial cells to form vascular-like structures, laying the groundwork for 3D vascularized tissue development. Similarly, studies by Kubota et al. showed that endothelial cells cultured on Matrigel, a laminin-rich ECM derived from mouse sarcoma, spontaneously formed capillary-like structures (88). This highlighted the importance of providing a physiologically relevant surface, rather than plastic surfaces, for accurate vascular modeling (Figure 3; Bottom) (65). These foundational observations have greatly influenced the development of current blood vessel tissues and organoids, which aim to replicate the complex architecture of vascular networks (64, 72, 89, 90). These advanced 3D culture systems often combine multiple cell types, such as pericytes and smooth muscle cells, with endothelial cells to promote the formation of more representative blood vessels, effectively bridging the gap from simple 2D cultures to complex, multi-cellular organoids.

Endothelial cells in culture exhibit specific properties that facilitate their identification and isolation. Voyta et al. showed that endothelial cells internalize and degrade acetylated-low-density lipoprotein (Ac-LDL) significantly more than smooth muscle cells or pericytes (91). This characteristic indicates their active role in lipid metabolism and vascular homeostasis. Crucially, it was exploited to distinguish endothelial cells using Ac-LDL conjugated with a fluorescent probe, enabling their isolation via fluorescence-activated cell sorting (FACS). Besides the uptake of Ac-LDL, other characteristics have been employed for endothelial cell identification. These include the expression of specific cell surface markers like CD31, CD34, and von Willebrand factor (vWF) (92). Another distinguishing feature is their capacity to form capillary-like structures *in vitro* on reconstituted basement membrane matrices like Matrigel (88). Furthermore, endothelial cells exhibit distinct intercellular junctions visible under electron microscopy and specifically bind certain lectins, such as Ulex europaeus agglutinin I (UEA-I) (93). Utilizing these combined phenotypic and functional characteristics ensures accurate identification and isolation of endothelial cells for vascular tissue and organoid engineering.

The long-term culture of endothelial cells is highly desirable for *in vitro* studies, especially for developing vascularized tissue and organoid models. Folkman et al. successfully achieved the long-term culture and cloning of capillary endothelial cells from various sources, including human and bovine tissues (94). Prior to their work, maintaining capillary endothelial cells *in vitro* for extended periods was challenging, as these cells typically failed to grow beyond a few weeks. By modifying existing methods and utilizing tumor-conditioned medium, gelatin-coated plates, and techniques for enriching capillary endothelial cells in primary culture, the team managed to culture cells from multiple human and animal tissues. This breakthrough laid the foundation for further research on endothelial cell cultures and their growth and survival *in vitro*. Researchers have made notable strides in enhancing the long-term culture of endothelial cells (95–97). Current culture techniques now employ specialized growth mediums, such as EGM-2, which contains growth factors like VEGF, FGF, and EGF, ensuring a chemically defined, nutrient-rich environment that supports cell proliferation and longevity (98–100). The advent of 3D culture systems, including vascular spheroid and organoid cultures, has enabled more natural growth and interactions with vascular cells (74). Furthermore, advanced mechanical approaches, such as bioreactors and microfluidic technologies, have also been instrumental in promoting the long-term viability of endothelial cells in vascularized tissues and organoids.

Bioreactors are vital in mimicking blood flow and crucial for maintaining endothelial cell functionality and morphology over extended periods. This is particularly relevant in vascularized tissues and organoids, where simulating the dynamic environment of blood vessels is crucial for accurately replicating physiological conditions (101). Similarly, integrating microfluidic systems offers a platform for creating more physiologically relevant shear stress, essential for preserving endothelial integrity and function (102). A more detailed exploration of bioreactors and microfluidic systems in relation to tissue and organoid vascularization will be addressed in another section.

So far, we have briefly charted the evolution of endothelial cell research, encompassing early culturing techniques to the development of sophisticated 3D vascularized tissues. The pivotal role of endothelial cells in vasculogenesis and angiogenesis underscores their significance in vascular biology. Seminal work involving HUVECs and identifying essential growth factors like FGF and VEGF have been central to advancements in tissue engineering. These studies have elucidated endothelial cell proliferation mechanisms, propelling the field towards more intricate 3D modeling.

### 3.2 Incorporation of multiple cell types

Co-culturing endothelial cells with supporting cells, alongside angiogenic growth factors like VEGF and FGF-2, drives the self-organization into tissue-specific vascular structures. Recent efforts have involved the integration of mesenchymal stem cells (MSCs), mesodermal progenitor cells (MPCs), and macrophages (103). Each cell type confers distinct advantages to the vascularization and functional maturation of organoids. MSCs have pro-angiogenic secretomes rich in factors like TGF-β and PDGF, promoting endothelial sprouting and stabilizing vascular networks (104). MPCs differentiate into various mesodermal derivatives, including pericytes and smooth muscle cells, contributing to the structural integrity and functionality of the vascular system (105). Macrophages play dynamic roles in angiogenesis, tissue remodeling, and innate immunity (106). Thus, including these cells in vascularized tissue models introduces a critical aspect of the immune-vascular interface and enhances the mimicry of the *in vivo* tissue microenvironment.

MSCs have garnered interest due to their multipotency, secretion of angiogenic and trophic factors, and potential for differentiation into mural cells like pericytes and vascular smooth muscle cells (104). Mural cells play a vital role in vessel assembly, stability, and maturation (107). Pericytes envelope endothelial tubes and are essential for vessel maturation and hemodynamic stability (108). Furthermore, incorporating mural cells provides structural and mechanical support akin to native vessels (109). These cells contribute signaling cues and ECM components, guiding vessel formation and integrity (109–111). The importance of mural cells extends to developing and maintaining the blood-brain barrier (BBB) (112). Consequently, they are vital in modeling diseases where BBB integrity is compromised, including Alzheimer's disease (113), seizure (114), and stroke (115). The pro-angiogenic role of MSCs has been studied in other contexts, including tissue regeneration in avascular scaffolds. In their study, Summer et al. showed that co-cultured MSCs and HUVECs formed interconnected vascular networks within avascular human platelet lysate-based matrices (116). Besides directly promoting angiogenesis, MSCs can indirectly mediate the *in vitro* pro-angiogenic effects of factors like tropoelastin (117).

MPCs are integral to advancing tissue and organoid vascularization due to their capacity to differentiate into various cell types, including endothelial, vascular smooth muscle, and hematopoietic cells (105). This differentiation spectrum is essential for replicating complex vascular architecture within organoids. Dogan et al. showed the potential of hPSC-derived MPCs (hiMPCs) in bioprinting realistic vascular systems (118). When these cells were formulated into an alginate/ collagen bioink and extruded, they mimicked embryonic vasculogenesis, forming hierarchical vessels with multilayered walls. Furthermore, MPCs have been used to generate functional organoids. Wörsdörfer et al. described the integration of iPSC-derived MPCs into organoids to create complex vascularized human tumors and neural organoids (119). The MPCs contributed to forming hierarchically organized and structurally detailed blood vessel networks that showed dynamic growth and response to angiogenic stimuli. Importantly, these bioengineered vessels anastomosed with host vasculature upon transplantation, which is a crucial step for integration and functionality in a living organism. These findings underscore the pivotal role of MPCs in enhancing the physiological relevance and potential clinical utility of organoid models.

Finally, due to their significant role in angiogenesis, incorporating macrophages into vascularized tissues is necessary for modeling diseases like cancer, atherosclerosis, and microbial infections. Consequently, macrophages have been co-cultured with endothelial cells to serve as support cells in vascularization (106). When polarized towards a pro-inflammatory profile, macrophages significantly increase the number and length of endothelial sprouts, mediated through Notch signaling (120). Many studies have highlighted the potential of macrophage phenotypes in enhancing vascularization (121). Moore et al. found that encapsulating endothelial cells with M2 and M0, but not M1, macrophages in a bioactive hydrogel significantly increased the formation of vascular tubules (122). Earlier, Spiller et al. challenged the conventional view of the role of macrophages in angiogenesis, demonstrating that both M1 and M2 macrophages contribute to this process, each playing distinct roles in vascular development and remodeling (123). The role and interactions of macrophages with tissue-specific vascular cells are still an active research area. The incorporation of macrophages in vascularized tissues and organoids can be complicated by their highly plastic nature and the influence of biomaterials on macrophage behaviors (121).

### 3.3 Incorporation of organotypic endothelial cells and tissue-specific cues

To improve the capacity of vascularized tissues and organoids to recapitulate the native vascular niche accurately, multiple cell types began to be incorporated into the culture systems. The pioneering work of Levenberg et al. provided a novel approach by co-culturing endothelial cells with other cell types, such as fibroblasts and skeletal muscle cells, to form 3D vascularized skeletal tissues (124). Rouwkema et al. and Kyriakidou et al. carried out similar work involving bone tissue engineering (125, 126). These studies demonstrated the potential of co-culturing endothelial cells with other cell types to form vascularized tissues.

Endothelial cell sources are typically from human umbilical vein endothelial cells (HUVECs), endothelial progenitor cells (EPCs), or induced pluripotent stem cells (iPSC-ECs) (12, 13, 127, 128). iPSC-ECs are noninvasively derived from human induced pluripotent stem cells and will be covered in a different section of the review. EPCs play critical roles in postnatal endothelial repair and neovascularization of ischemic organs (129). For vascular tissue engineering, they can be derived from adult peripheral and umbilical cord blood (127). They can also be derived from the bone marrow (130). However, there are challenges with identifying and characterizing EPCs and selecting the appropriate culture conditions for EPC microvessel formation (127). Consequently, they are not often the primary choice of researchers for *in vitro* vascularization. As previously discussed, due to their commercial availability and ease of isolation and culture, HUVECs are widely used in tissue engineering to study endothelial cell behavior and vascularize *in vitro* models (131).

Despite being commonly used in tissue and organoid vascularization, HUVECs differ from the endothelial cells in different tissues. Endothelial cells generally exhibit remarkable heterogeneity in their structural and functional attributes (132). This heterogeneity is shaped by the cellular microenvironment, growth factor response, and organ-specific regulatory elements (133). Recent advancements in single-cell RNA sequencing (scRNA-seq) have unveiled distinct transcriptomic profiles in endothelial cells across different organs, providing insights into the molecular underpinnings of this heterogeneity (134). Marcu et al. investigated endothelial cells (ECs) from developing heart, lung, liver, and kidneys, uncovering distinct gene expression patterns and cellular functions specific to each organ (135). These findings demonstrate the vital role of organ-specific endothelial cells in developmental processes and their potential in applications like organ regeneration, disease modeling, and differentiation from hPSCs. This review will briefly highlight the characteristics of brain microvascular endothelial cells (BMECs), liver sinusoidal endothelial cells (LSECs), and cardiac microvascular endothelial cells (CMVECs). A comprehensive review can be found in Nguyen et al. and Trimm et al. (136, 137).

The brain requires a highly selective barrier to tightly regulate the influx of substances and maintain a stable internal environment. This barrier, known as the blood-brain barrier (BBB), is formed by BMECs, which are connected by tight junctions and further reinforced by astrocyte end-feet (138, 139). The BBB has several transport systems, including efflux transporters, for the selective permeation of essential molecules and preventing the entry of toxic substances (140). Functionally, BMECs have high transendothelial electrical resistance (TEER), indicating tight regulation of transport (141). This resistance is influenced by several factors, such as astrocytes (142), the viscosity of the culture medium, and electrode type (143). BMECs express high levels of claudin-5 and occludin, involved in tight junction formation (144); ZO-1, a tight junction protein (145); GLUT-1, a glucose transporter; and p-glycoprotein, an efflux transporter.

Conversely, the liver demands a more permeable vasculature for efficient filtration and metabolism, thus requiring LSECs, which form a highly permeable barrier (146). Accordingly, LSECs are characterized by large fenestrations and discontinuous basement membranes. LSECs display a range of receptors and adhesion molecules, such as stabilin-1 (STAB1), stabilin-2 (STAB2), lymphatic vessel endothelial hyaluronan receptor-1 (LYVE1), and thrombomodulin (THBD), crucial for scavenging functions, the uptake of macromolecules from the blood, and other liver-specific functions (147). Moreover, they also regulate hepatic microcirculation, maintain immune homeostasis, and contribute to liver fibrosis and regeneration (148, 149). LSEC dysfunction has been implicated in various liver disorders, including metabolic dysfunction-associated steatotic liver disease (MASLD) (formerly NAFLD) (150).

Finally, in the heart, CMVECs are constantly exposed to mechanical forces, including shear and cyclic stress, due to blood flow and vessel deformation (151). CMVEC responses to these mechanical stimuli involve alterations in cell morphology, cytoskeletal architecture, and overall function (152). CMVECs and cardiomyocytes engage in a dynamic, bi-directional crosstalk, essential for cardiac function and homeostasis (153). CMVECs secrete several paracrine factors, such as nitric oxide, prostaglandins, and endothelin-1, which modulate cardiomyocyte contractility, metabolism, and hypertrophic responses (154–156). Conversely, cardiomyocytes influence CMVECs by secreting VEGF, which promotes coronary angiogenesis. Disruptions in these interactions are implicated in heart failure and ischemic heart disease. Advanced transcriptomic analyses by Litvinukova et al. unveiled the heterogeneity in CMVECs (157). The team identified ten distinct populations of endothelial cells in the heart and three significant types of capillary endothelial cells, which comprise over half of all CMVECs and express *RGCC* and *CA429*. They also found other specialized CMVEC populations.

In engineering physiologically relevant vascularized tissues and organoids, several research studies have demonstrated the importance of providing organotypic signaling cues. This has been achieved using organ-specific microenvironmental cues or organ-specific vascular cells. Lippmann et al. generated BBB-like properties in hiPSC-ECs by exposing the cells to retinoic acid and co-culturing them with neural progenitor-derived astrocytes and pericytes (158). The team later published a more cost-effective protocol for generating human BMECs involving the use of Essential 6 (E6) medium (159), which was subsequently modified by Pong et al. (160) for the expansion and cryopreservation of BMECs. Human BMECs have also been generated from hPSC-derived endothelial progenitor cells (161) and the BC1 cell line (162). Besides the co-culture approach, BMECs have been generated from hPSCs by activating specific signaling pathways using small molecules (163).

**Figure 4:**
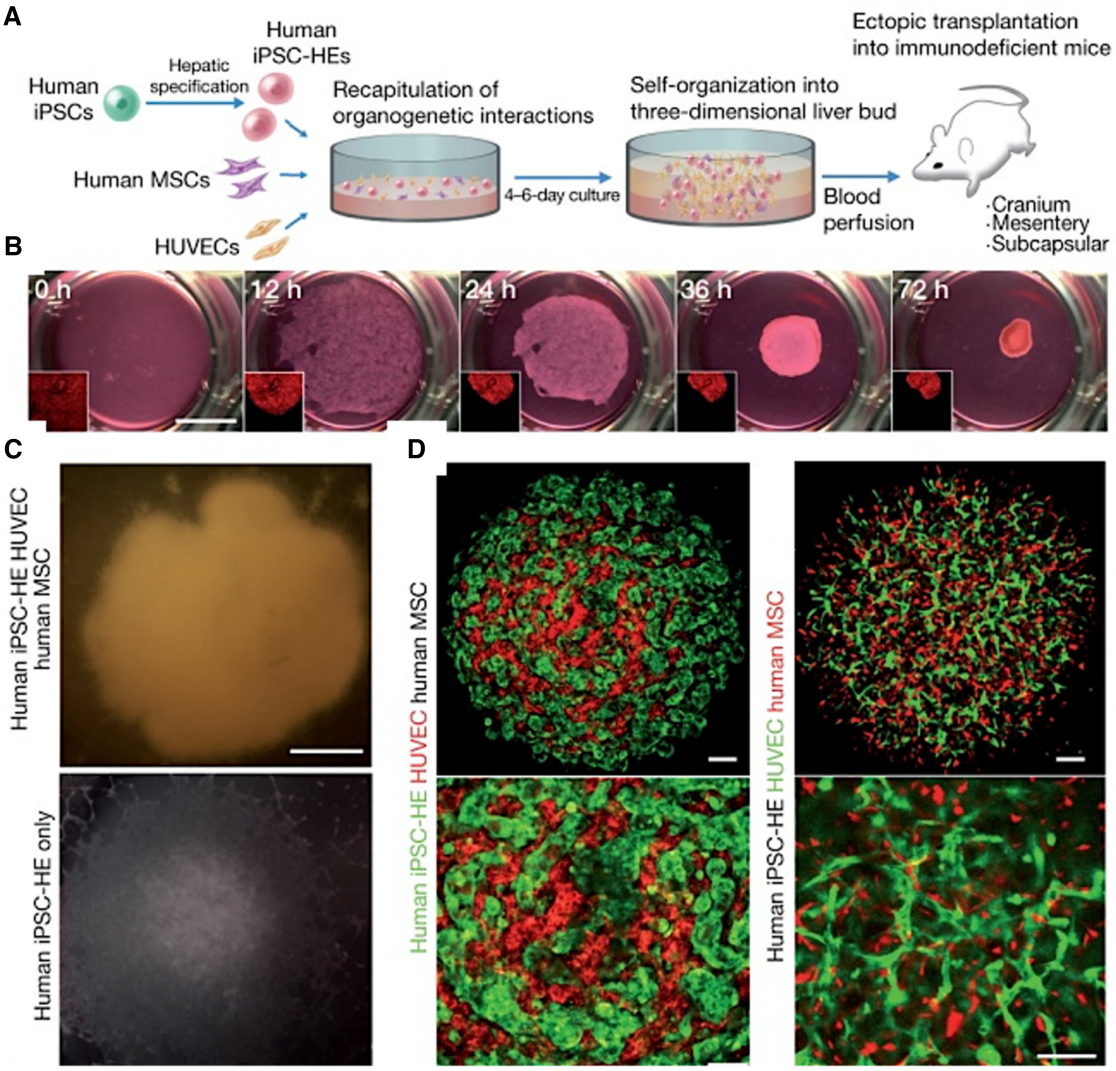
Generating vascularized liver buds using iPSCs. (**A**) Starting with human iPSCs, hepatic specification is induced, followed by the introduction of human mesenchymal stem cells (MSCs) and HUVECs. This results in self-organization into a 3D liver bud structure, which is subsequently transplanted into mice for further development and blood perfusion. (**B**) The progression of liver bud formation over time in culture, starting from a homogenous cell mixture at 0 h and progressing to a distinct, spheroid liver bud structure by 72 h. (**C**) Comparing the gross appearance of liver buds with and without HUVECs demonstrates the critical role of HUVECs in the formation of liver buds. (**D**) Via confocal microscopy, the top images show the intricate network of HUVECs (stained red (left) or green (right)) intertwined with hMSCs (black (left) or red (right)), indicating successful co-culture and the establishment of a vascularized construct. The bottom images are enlarged areas of the top images. Used with permission from Takebe et al. (164) Copyright © 2013, Springer Nature Limited.

The co-culture system has been shown to promote the expression of tissue-specific markers and the maturation of vascularized *in vitro* systems. In a seminal study, Takebe et al. demonstrated the successful vascularization of liver buds by co-culturing human iPSC-derived hepatic endoderm cells, mesenchymal stem cells, and HUVECs (Figure 4). This triculture system led to the spontaneous formation of vascular-like structures within the liver buds (164). The researchers did not characterize the vessels for LSEC markers but noted that the vessels were functional and improved the maturation of the liver buds (164). A similar approach was used by Sato et al. to generate vascularized human placenta from iPSC-derived organ bud transplant (165). The interactions between iPSC-derived cardiomyocytes and iPSC-derived endothelial cells have been explored in a co-culture system. Helle et al. showed that, within 48 h of co-culture, iPSC-derived endothelial cells began to exhibit characteristics of cardiac-specific endothelial cells, with increased maturity and homogeneity (166). Similarly, including LSECs in liver organoids has been shown to enhance the structural and functional maturation of the organoids (167, 168). Yap et al. successfully developed vascularized hepatobiliary organoids by co-culturing liver progenitor cells (LPCs) with LSECs (167). In contrast to LPC-only organoids, which showed mild hepatobiliary differentiation, LPC/ LSEC organoids exhibited significant hepatocyte-like cell formation, biliary duct development, and upregulation of hepatic and biliary genes within seven days (167).

Recent advances in tissue and organoid vascularization, highlighted by Lippmann et al., Takebe et al., and others, emphasize the critical role of organotypic signaling and co-culture systems in mimicking specific organ properties and vascular networks. These techniques have improved the physiological relevance of vascularized systems and facilitated their scalable and cost-effective production. The integration of various cell types has led to vascularized tissues and organoids with enhanced maturity and functionality, as demonstrated in liver and brain models. This progress marks a significant stride in developing complex, physiologically accurate models for drug discovery and disease modeling.

### 3.4 Human pluripotent stem cells in vascular modeling

Human pluripotent stem cells (hPSCs) include human embryonic stem cells (hESCs) and human induced pluripotent stem cells (hiPSCs). Remarkably, somatic cells can be reprogrammed into human induced pluripotent stem cells (hiPSCs) using Yamanaka factors (*OCT3/4*, *SOX2*, *KLF4*, *c-MYC*) (169). This breakthrough has allowed for the creation of patient-specific hiPSCs, crucial in personalized medicine. The potential of hiPSCs to differentiate into virtually any cell type, including endothelial and stromal cells, offers the opportunity to generate complex, physiologically relevant tissues. In this section, we explore the strides made over the past few decades, examining a few seminal protocols developed to differentiate hPSCs into vascular cell types.

Embryoid body (EB) formation and monolayer-directed differentiation represent two distinct methods for stem cell differentiation, each with unique advantages and challenges (170, 171). EB formation, a 3D aggregation technique, mimics early embryonic development, promoting spontaneous differentiation into multiple lineages (170, 172). This approach, while efficient in generating diverse cell types, often leads to heterogeneous populations, posing challenges in reproducibility and precise lineage control. Conversely, monolayer-directed differentiation, where cells grow in a 2D format, allows for tighter control over the microenvironment, including the application of specific growth factors (173). This method enhances reproducibility and specificity in generating desired cell types but may require intricate protocols to achieve the same breadth of differentiation as EBs. For tissue vascularization, EBs may offer a more physiologically relevant model for studying vasculature development due to their 3D structure (174). However, the differentiation of hPSCs into EBs (including vascular cell types) is inefficient (1%–5%). Monolayer techniques provide a simplified platform for investigating molecular mechanisms in endothelial cell differentiation and function. They are relatively more efficient (5%–20%) and may rely on undefined supplements or conditioned media, limiting their reproducibility (175). In the past decade, several seminal protocols involving monolayer-directed differentiation in chemically defined media have moved the field forward. Some of these works will be discussed in this section.

In a pivotal study, Yamashita et al. differentiated Flk1+ cells derived from embryonic stem cells into endothelial and mural cells (176). The researchers demonstrated that VEGF stimulates endothelial cell differentiation, while PDGF-BB induces mural cell formation. They showed that the Flk1+ cells can form vessel-like structures in 3D culture and integrate into the developing vasculature *in vivo* when injected into chick embryos. Levenberg et al. observed a significant increase in the expression of various endothelial genes during the differentiation of EBs, with the highest expression levels occurring between days 13 and 15 (177). The researchers isolated embryonic PECAM1-positive cells, demonstrating that these cells in culture exhibit characteristics akin to vessel endothelium, including forming tube-like structures on Matrigel. Building on this foundation, Ferreira et al. identified a population of vascular progenitor cells (CD34+) within hESCs that differentiate into endothelial-like and smooth muscle-like cells when cultured with VEGF-165 and PDGF-BB, respectively (178). A significant milestone in the field was the simultaneous differentiation of vascular progenitor cells into endothelial cells and SMCs by Hill et al. (179).

In 2012, Dar et al. published one of the earliest protocols detailing the derivation of vasculogenic pericytes from hPSCs. The team used a combination of common and specific vascular cell markers to investigate the emergence of pericytes alongside endothelial and smooth muscle cells (180). They identified a novel population of cells positive for pericyte markers (NG2, PDGFR-β, CD146) but not for α-SMA, displaying characteristics of mesenchymal stem cells. The absence of α-SMA suggests that these hPSC-pericytes are immature (181), which may limit their physiological relevance. Co-implanting the hPSC-derived pericytes with endothelial cells led to functional integration into mice vasculature, enhancing vascular and muscle regeneration in the murine model with limb ischemia. Notably, Dar et al. showed that hPSC-pericytes are multipotent, as evident by their ability to differentiate into osteoblasts, chondrocytes, adipocytes, and muscle cells under specific conditions (180). The multipotential nature of pericytes has implications for vascular remodeling and makes them essential for modeling vascular pathologies *in vitro*.

**Figure 5:**
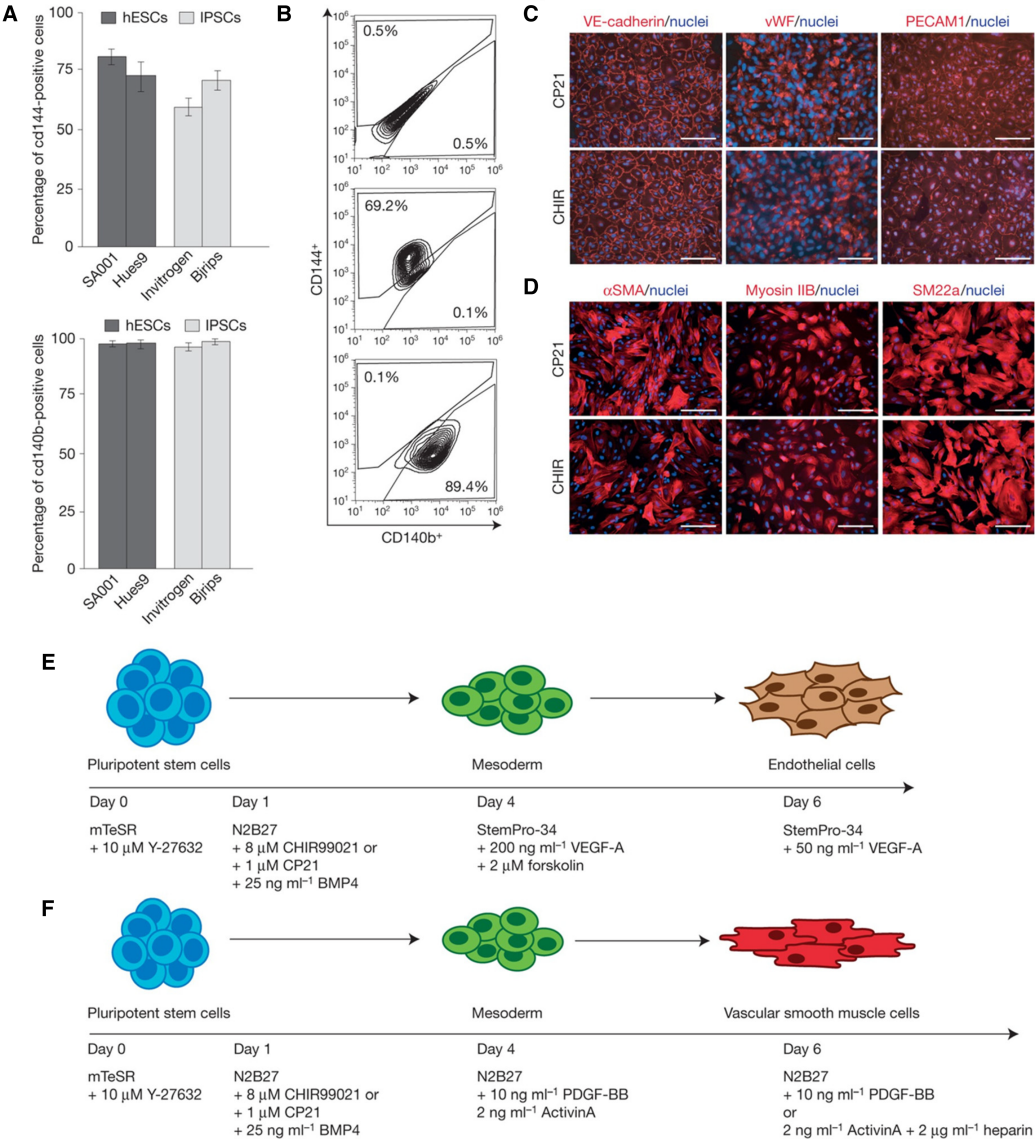
Differentiating hPSCs into ECs and SMCs. (**A**) Bar graphs comparing the efficiency of differentiation into vascular endothelial (VE) cells from hESCs and iPSCs under various conditions. (**B**) Flow cytometry plots providing cell surface marker expression (CD144+, CD140b+) indicative of VE cells, demonstrating the purity of the cell populations resulting from the differentiation process. (**C**) The expression of VE-cadherin, vWF, and PECAM1 demonstrating the presence of endothelial cells. (**D**) The expression of αSMA, myosin IIB, and SM22α demonstrating the presence of vascular smooth muscle cells. (**E**) Stepwise differentiation protocol from pluripotent stem cells to mesoderm and then to endothelial cells, including the specific growth factors and inhibitors used at each stage, such as BMP4, CHIR99021, VEGF-A, and others. (**F**) Protocol for differentiating pluripotent stem cells into vascular smooth muscle cells, detailing the sequential addition of growth factors and signaling molecules like Activin A, BMP4, and heparin. Used with permission from Patsch et al. (175) Copyright © 2015, Springer Nature Limited.

Several protocols to generate vascular and perivascular cells from progenitor cells and hPSCs have been published in the last decade. Kurian et al. converted human fibroblasts to angioblast-like progenitor cells capable of being further differentiated into endothelial and smooth muscle lineages (182). Orlova et al. published a seminal study demonstrating the simultaneous differentiation of hPSCs into endothelial and pericytes/mural cells (183). The team induced mesodermal lineage by activating the Wnt signaling pathway using Activin A, BMP4, VEGF, and CHIR99021, followed by vascular specification using VEGF and SB431542 (a TGF-β inhibitor). Remarkably, endothelial cells and pericytes were isolated by day 10. While this protocol involved a chemically defined medium and was reproducible across several hPSC lines, significant variations were observed, and the efficiency was relatively low (10%–30%). Patsch et al. developed a rapid and efficient method for differentiating hPSCs into vascular endothelial and smooth muscle cells (175). This process involved initial GSK3 inhibition and BMP4 treatment to direct cells toward a mesodermal fate (Figure 5). This was followed by exposure to either VEGF-A for endothelial cells or PDGF-BB for vascular smooth muscle cells, achieving over 80% efficiency within six days. These improvements allow modeling patient-specific vascular pathologies using autologous hPSCs rather than relying on HUVECs and EPCs.

Wimmer et al. introduced a landmark protocol to produce self-assembling human blood vessel organoids from hiPSCs (64). These vascular organoids exhibited a complex architecture, with branching capillaries and endothelial cells expressing markers characteristic of human blood vessels, further enriched by pericytes. Remarkably, these organoids demonstrated susceptibility to hyperglycemic conditions, mimicking diabetic vasculopathy phenotypes. Nonetheless, the model lacked interactions with other somatic cell types, including immune cells, potentially limiting their efficacy in studying organ-specific diseases. Additionally, they could not form larger, more complex vessels such as arterioles or venules *in vitro*. Another potential drawback was the introduction of batch variability due to the collagen I-Matrigel matrix. More recently, Schmidt et al. offered a novel approach to model the early and late phases of human blood vessel development (72). Unlike the protocol by Wimmer et al., their approach involved a single administration of VEGF and excluded supplementation with FGF-2 and forskolin. Crucially, hiPSCs were suspended in non-adhesive agarose-coated wells to form 3D aggregates, obviating the need for a Matrigel matrix. However, the model had few mural cells *in vitro*.

The derivation of endothelial cells from hPSCs has enabled studying endothelial functionality and pathology under controlled conditions. Crucially, the advent of patient-specific hPSCs has particular significance for modeling genetic vascular diseases. hPSCs derived from patients with specific vascular disorders can be induced to differentiate into vascular cells, thus generating “disease in a dish” models that carry the disease's genetic aberrations and phenotypic characteristics. For example, Atchison et al. generated a tissue-engineered vascular model of Hutchinson-Gilford progeria syndrome (184). Moreover, modeling vascular diseases using hPSCs extends to complex disorders such as diabetes, where endothelial dysfunction plays a role. Using hPSC-derived endothelial and mural cells, researchers can investigate the influences of hyperglycemia on microvascular complications (64).

Next, we focus on applying hydrogels as ECM mimics in vascular tissue and organoid engineering. Due to their structural and compositional resemblance to the ECM, hydrogels present a unique opportunity to recreate the complex endothelial microenvironment. Their use in 3D culture systems is not merely a technological advancement but a paradigm shift in how we approach the engineering of vascularized constructs. We will examine the properties of hydrogels that make them ideal for such applications, their integration into current vascular engineering strategies, and how they transform our understanding of endothelial cell behavior and function.

### 3.5 Hydrogel scaffolds as ECM mimics

The late 20th century marked a turning point in the evolution of tissue vascularization, recognizing the ECM as a critical determinant in cell behavior, tissue organization, and fate decisions (185). This realization spurred developing more sophisticated *in vitro* models, with hydrogels emerging as a pivotal innovation. As 3D biomaterials, the crosslinked networks of hydrophilic polymers in hydrogels closely mirror the physical properties of the ECM. Their porosity enables substantial absorption of biological fluids, which simulates the hydration and nutrient diffusion dynamics of *in vivo* ECMs (186).

Endothelial cells within hydrogel scaffolds transduce mechanical stimuli from the microenvironment of the hydrogels into biochemical responses, a process pivotal for vascular development and function (187). The mechanical properties of hydrogels, including stiffness and elasticity, have been shown to exert profound influences on endothelial cell behavior. Turturro et al. demonstrated that hydrogels with lower stiffness promote enhanced endothelial sprouting and microvascular network assembly (188). Lampi et al. provided insights into how endothelial cells respond to variations in matrix stiffness and highlighted the influence of mechanical heterogeneity in vascular tissue engineering (189). Beyond mechanical properties, the chemical composition and degradation kinetics of hydrogels dictate cell migration and differentiation. A model developed by Liu et al. showed that endothelial cell sprouting and the formation of perfusable blood vessels within a hydrogel are dependent on the adhesiveness and degradability of the hydrogel (190). These properties need to be finely balanced to support the initial invasion of endothelial cells and subsequent matrix remodeling for lumen formation.

Natural hydrogels like alginate, collagen, and fibrin have intrinsic biocompatibility, making them the preferred scaffolds for many early researchers. They were used to investigate angiogenesis (191), cell proliferation (192), and differentiation (193). Collagen is the most abundant protein in the mammalian ECM. Its fibrillar structure imparts mechanical strength and provides important cues for cellular attachment, proliferation, and differentiation, essential for vasculogenesis (194). Collagen-based hydrogels have been extensively used to model vascular ECM due to their biocompatibility and bioactivity. Gelatin, a denatured form of collagen, retains many of the cell-interactive properties of collagen (195). Functionalized gelatin, such as gelatin methacryoyl (GelMA), has also gained prominence in vascular tissue engineering (196, 197). Its photolinkable property allows for precise control over mechanical properties and degradation rates, facilitating neovessel formation. Hard tissue applications like bone regeneration use stiffer hydrogels, such as modified gelatins or collagen composites (198, 199). These hydrogels provide the necessary rigidity and support for osteogenesis while facilitating vascularization. Fibrin is another key ECM component. It functions in wound healing and angiogenesis. Fibrin hydrogels mimic the natural wound matrix, supporting endothelial cell migration and tubulogenesis (200, 201). Alginate and hyaluronic acid are polysaccharides in the ECM and offer unique advantages in tissue vascularization (202). Alginate is a naturally occurring biopolymer notable for its mild gelation conditions and ability to encapsulate cells with minimal damage (203). Hyaluronic acid hydrogels have been employed to study the interactions between endothelial cells and the vascular basement membrane (204). In soft tissue engineering, such as cardiac or hepatic tissues, alginate, and hyaluronic acid hydrogels are favored for their soft, elastic properties, closely mimicking the natural tissue environment (205, 206). They support the formation of delicate vascular networks essential for the functionality of these organs.

Natural hydrogels, while advantageous for their biocompatibility and bioactivity, have several limitations. Their inherent variability, stemming from batch-to-batch differences, can lead to inconsistent cell responses, complicating standardization in tissue engineering applications (207). Moreover, these hydrogels often exhibit inadequate mechanical strength and have unpredictable degradation rates (208). As a result, they are not always conducive to the formation and maintenance of stable, complex vascular networks. Limited customizability is another critical drawback. Natural hydrogels offer a restricted scope for chemical modification to precisely control properties like porosity, bioactive signaling, and degradation kinetics (209). These modifications are crucial for orchestrating the intricate process of tissue growth and vascularization. Additionally, certain natural hydrogels may trigger immune responses, potentially compromising the integration and functionality of the vascularized tissue (209). To improve their properties, researchers have employed biofabrication techniques, such as 3D bioprinting and soft-nanoparticle functionalization (210, 211). Nanocomposite hydrogels offer higher mechanical strength, emerging as a promising technology in tissue engineering (212). However, there are challenges to understanding the nano-/micro-structure of these hydrogels and their interactions with cells (208). Another bioengineering approach to improving mass transport and cellular viability within natural hydrogels is 3D micromolding (213). This technique involves the fabrication of microchannel networks within hydrogel constructs.

The advent of synthetic hydrogels marked a significant leap forward in tissue and organoid engineering. These hydrogels are synthesized by various chemical methods and functionalized with bioactive molecules. As a result, they offer customizable biomechanical platforms that have enabled researchers to precisely mimic the dynamic landscapes of native tissues. Polyethylene glycol (PEG)-based hydrogels are widely used in tissue vascularization due to their hydrophilicity, resistance to protein and cell adsorption, and biocompatibility (214). The capacity of PEG hydrogels to encapsulate bioactive molecules, such as VEGF (215) and arginylglycylaspartic acid (RGD) (216), enhances endothelial cell migration and proliferation. They also support co-culture systems, accommodating endothelial and perivascular cells (217). Other synthetic hydrogels include polylactic acid (PLA), poly(lactic-co-glycolic acid) (PLGA), and polycaprolactone (PCL). In tissue vascularization, these hydrogels are often combined with natural hydrogels to form hybrid (composite) hydrogels (218).

Hybrid hydrogels synergize the biocompatibility of natural materials with the mechanical precision of synthetic polymers. Jiang et al. created hybrid hydrogels combining porous PEG scaffolds and fibrin within the pores to promote vascularized tissue formation (219). Using a salt leaching technique for the porous structure and preloading with thrombin, the hydrogels facilitated fibrin polymerization. Notably, the hybrid hydrogel demonstrated enhanced tissue invasion and vascularization in a rodent model compared with hydrogels without fibrin. Jung et al. developed a more recent fibrin-based composite hydrogel, combining fibrin and dextran-methacrylate (MA) (220). A detailed exploration of the different hydrogels (natural, synthetic, and composites) has been published by Yeo et al. (218) and Barrs et al. (214).

Researchers have designed stimuli-responsive hydrogels that can interact with their microenvironments to better model the dynamic nature of physiologic and pathological processes (221). These so-called “smart” hydrogels can respond to environmental stimuli such as temperature, pH, light, and proteins. Smart hydrogels are poised to revolutionize tissue and organoid vascularization by offering controlled release of growth factors and aligning more closely with the complexities of the natural ECM in vascularized tissue development (222). They mimic the physical, mechanical, and biological properties of natural tissues, providing an ideal environment for cell growth, 3D structure support, and delivery of bioactive molecules. Poly(vinyl alcohol) (PVA) methacrylate, a photopolymerizable hydrogel, has been utilized for its spatial patterning capabilities, which allow for the creation of vascularized tissues with high-resolution structures (213, 223). Moreover, smart hydrogels have been used to improve bone repair, as shown by Yang et al. The team introduced a novel enzyme-sensitive hydrogel microsphere (KGE) for delivering bone marrow mesenchymal stromal cell-derived exosomes (BMSC-Exos) in response to neovessel formation during bone healing (224). Utilizing matrix metalloproteinase-1 (MMP1) responsive materials, these microspheres release exosomes in areas of neovascularized bone, promoting stem cell migration and osteodifferentiation. There have been other exciting applications of smart hydrogels in tissue engineering. El-Husseiny et al. published a comprehensive review detailing these latest developments (225).

### 3.6 Rise of three-dimensional (3D) bioprinting and ECM patterning

Vascularization is critical for ensuring tissue viability and function post-implantation, primarily by facilitating essential processes such as nutrient supply and waste removal. In *de novo* strategies for tissue vascularization, a critical limitation is the inability to consistently generate perfusable vascular structures. This challenge is compounded by a lack of precision in controlling the spatial organization of these vascular networks. Current methodologies often result in either non-functional or poorly integrated vessels with the host circulatory system. Furthermore, the random arrangement of these vessels leads to inefficient tissue perfusion and can compromise the functional integration and longevity of engineered tissues. Bioprinting has emerged as a bioengineering strategy to fabricate complex and perfusable vascular networks.

Bioprinting is a rapidly evolving technology used to create tissues and organoids with integrated vascular networks (226). This technology employs precise, layer-by-layer deposition of biological elements, including various cell types, to construct functional tissues. Such intricately designed architectures are pivotal for fabricating tissues that closely mimic natural organ structures (227). This advancement holds significant potential for addressing the organ donor shortage, possibly leading to customized and more compatible organ transplants. As bioprinting technology advances, it promises to mitigate this shortage and improve the functionality of replacement organs. However, replicating the full complexity of native tissues and ensuring the long-term viability of implanted organs are challenges that remain active research areas. This section briefly delves into various bioprinting modalities, highlighting their applications in constructing functional vascular networks, with an in-depth review by Barrs et al. (214).

Before initiating the bioprinting process, obtaining a high-resolution 3D image of the target vascular structure is critical (218, 228). Advanced vascular imaging technologies are essential in this phase, providing detailed and accurate representations of vascular architecture. Micro-computed tomography (micro-CT) offers high-resolution imaging capabilities, ideal for visualizing small blood vessels (229, 230). Magnetic resonance angiography (MRA) is notable for its non-invasive nature and ability to penetrate deep tissues without contrast agents (231, 232). Optical coherence tomography (OCT) provides real-time imaging with micrometer-level resolution, essential for capturing the fine details of vascular structures (233, 234). Other techniques, such as confocal microscopy and ultrasound-based imaging, contribute valuable insights. These imaging technologies are integrated into the bioprinting workflow, aiding in the design and planning phase to ensure the anatomical accuracy and functional relevance of the bioprinted vascular networks.

#### 3.6.1 Laser-assisted bioprinting (LAB), extrusion-based bioprinting (EBB), droplet-based bioprinting (DBB)

**Figure 6:**
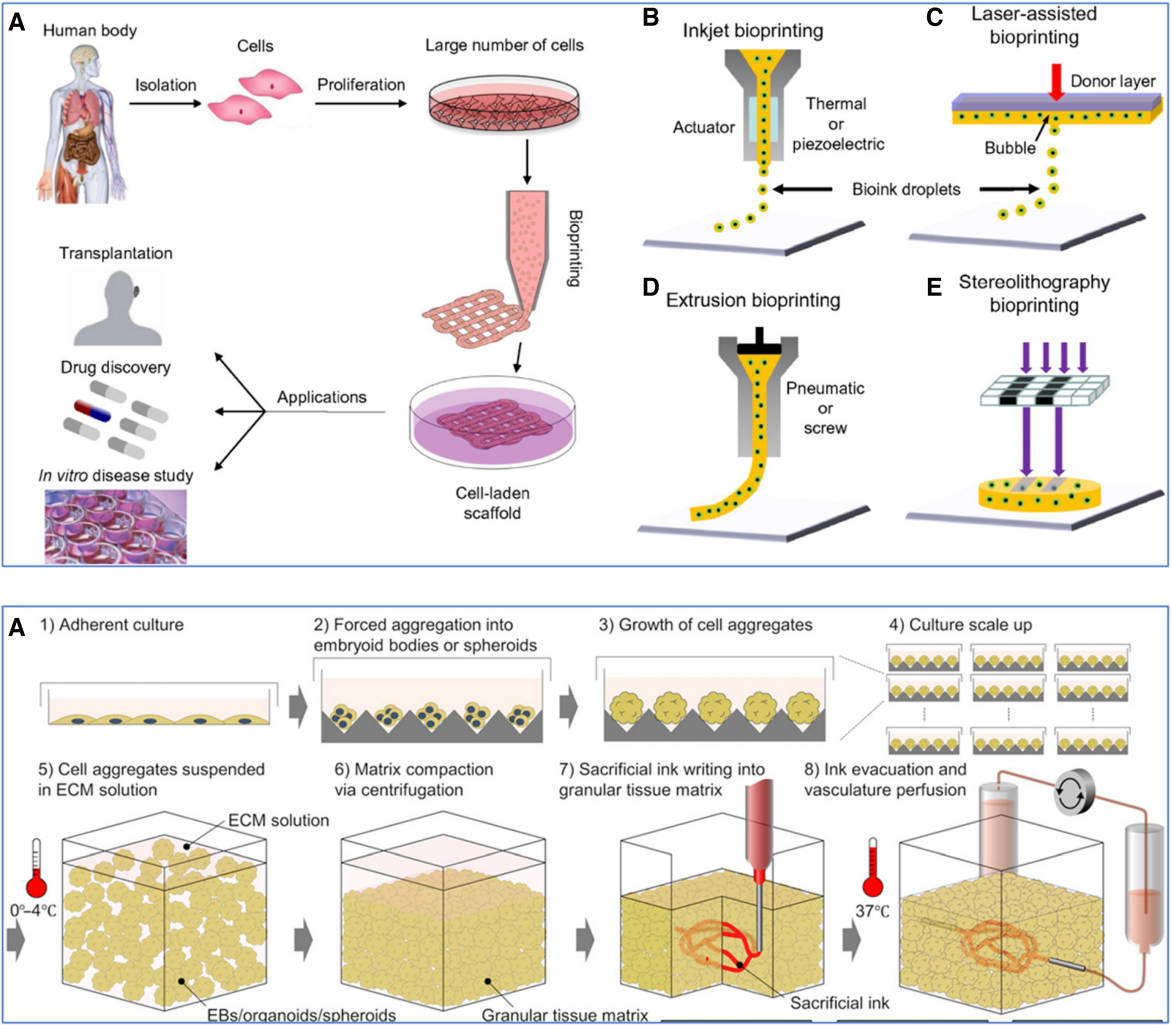
Various 3D bioprinting techniques. (**Top**): (**A**) Workflow of using cells from the human body. Initially, cells are isolated and then cultured to proliferate and create a large number of cells. These expanded cells can be used to develop cell-laden scaffolds through bioprinting, which can be applied in various domains such as transplantation, drug discovery, and *in vitro* disease study. (**B**) Inkjet bioprinting*,* also known as Droplet-Based Bioprinting (DBB), is where bioink droplets are deposited onto a substrate through an actuator, which can be either thermal or piezoelectric. (**C**) Laser-Assisted Bioprinting (LAB) uses a laser pulse to deposit a donor layer of bioink onto a substrate, creating a precise pattern of cells and scaffold material. (**D**) Extrusion-Based Bioprinting (EBB) employs a pneumatic or screw-driven mechanism to continuously deposit strands of bioink, building up a 3D structure layer by layer. (**E**) Stereolithography (SLA) Bioprinting, where light is used to selectively cure layers of a photo-sensitive bioink, stacking them to form a 3D structure. Used with permission from Mandrycky et al. (235) © 2015 Elsevier Inc. All rights reserved. (**Bottom**): Workflow for sacrificial writing into functional tissue (SWIFT). (**A**) 1: Cells cultured in an adherent manner, forming a monolayer on a flat surface. 2: These cells are then induced to aggregate, forming embryoid bodies or spheroids. 3: The cell aggregates continue to grow, increasing in size and complexity. 4: The process is scaled up, with multiple cell aggregates being cultured simultaneously. 5: The cell aggregates are then suspended in a cold (0–4°C) (ECM) solution to provide a supportive scaffold that mimics the natural cellular environment. 6: Centrifugation compacts the matrix, reducing the space between the aggregates and leading to a more dense and uniform tissue construct. 7: A sacrificial ink is introduced into the compacted tissue matrix. This ink is used to create channels within the matrix that will later form the vascular network. 8: The sacrificial ink is evacuated, leaving behind a network of hollow channels. The construct is then brought to physiological temperature (37°C), and a perfusion system is connected to these channels to simulate blood flow, allowing for the delivery of nutrients and oxygen to the tissue and the removal of waste products. Used with permission from Skylar-Scott et al. (236) licensed under CC BY-NC.

These are prominent 3D bioprinting modalities, each with unique mechanisms and applications in tissue vascularization (Figure 6; Top) (227, 235). LAB employs a focused laser pulse to deposit cell-laden materials onto a receiving substrate, facilitating high-resolution patterning (237). Its precision offers an advantage in creating complex tissue structures. Nonetheless, its reliance on sophisticated laser systems can be cost-prohibitive and limits accessibility. EBB, in contrast, extrudes bioinks through a nozzle, enabling the printing of larger tissue constructs with multiple cell types (16). This modality excels in fabricating bulk tissues but is often limited in achieving the fine resolution necessary for capillary-level vascular structures. Moreover, the mechanical stress during extrusion can also impact cell viability. DBB utilizes droplets of bioinks, striking a balance between resolution and viability (238). It allows for the deposition of cells and materials suitable for creating vascularized tissues. However, DBB faces challenges in achieving structural integrity and mechanical strength in larger constructs, as well as in precisely controlling droplet placement compared to LAB. LAB and DBB can be further subdivided into two approaches: indirect and direct bioprinting (18). Indirect bioprinting typically involves creating a mold or scaffold based on imaging data. In contrast, direct bioprinting involves the layer-by-layer deposition of cells and materials directly forming a vascular structure.

#### 3.6.2 Indirect 3D bioprinting

This technique has emerged as a strategy to tackle the challenge of creating complex, vascularized tissue constructs. This method involves using temporary, often biodegradable, sacrificial materials or fugitive bioinks integrated within the tissue matrix (16). These materials are removed following integration, leaving hollow channels that mimic natural vascular networks. Sacrificial materials commonly include water-soluble polymers or gels such as Pluronic F127, gelatin, and carbohydrate glass (239). These materials are selected for compatibility with living cells and ease of dissolution, ensuring minimal damage to the surrounding cellular environment. Pluronic F-127 has been used in several studies to create intricate vascular patterns due to its water solubility and structural integrity during printing (240–242). However, at high concentrations (10% w/w), Pluronic F127 can be cytotoxic (243). Indirect 3D bioprinting requires meticulous planning and sophisticated design, often leveraging advanced imaging and computational modeling to accurately replicate complex vascular architectures (218). However, this technique is limited by its resolution (>100 μm), which is much greater than the dimension of a typical capillary (5–10 μm) (214).

#### 3.6.3 Sacrificial bioprinting

This technique is a type of indirect 3D bioprinting, involves printing the fugitive bioink, which is later removed to create a network of channels within the tissue construct. These channels are subsequently endothelialized to form vascular-like structures. Compared to the self-assembly of endothelial cells, sacrificial bioprinting offers more control over the geometry of the fabricated lumens, allowing for the creation of complex and functional vascular networks (244). Miller et al. employed 3D-printed carbohydrate glass as a sacrificial template to create engineered tissues with perfusable vascular networks capable of supporting high-pressure blood flow and lined with endothelial cells (244). As Kolesky et al. demonstrated, sacrificial bioprinting can also be used to construct engineered tissues with integrated vasculature, various cell types, and ECM (242). The team printed with poly(dimethyl siloxane) (PDMS) and Pluronic F127-based inks to create vasculature and GelMA for the ECM and cell encapsulation. Although this work was a significant leap forward, the vascular networks were non-perfusable, limiting the construct's size. To address this limitation, Kolesky et al. (2016) developed a multi-material bioprinting method blend to create thick (>1 cm) vascularized human tissues capable of over six weeks of perfusion on a chip (245). The cell-laden inks contained a gelatin-fibrinogen blend, and Pluronic F127 was used as a fugitive bioink, both utilized at ambient conditions during the printing process.

#### 3.6.4 Sacrificial writing into functional tissue (SWIFT) bioprinting

This technique is another indirect bioprinting method that addresses the challenge of vascularizing thick tissue constructs. Developed by Skylar-Scott et al., SWIFT uses living organ building blocks (OBBs) matrices with suitable self-healing and viscoplastic properties to create high-density, functional organ-specific tissues (Figure 6; Bottom). The process involves compacting iPSC-derived OBBs into a matrix via centrifugation (236). A sacrificial bioink, typically composed of materials like gelatin, is printed directly into the densely populated cellular matrix. This sacrificial material forms a lattice that replicates the structure of the vascular network. Once the printing process is complete, the sacrificial material is gently removed, leaving behind a network of microchannels. These channels are subsequently seeded with endothelial cells to create a perfusable vascular system. The precision of SWIFT bioprinting allows for creating highly complex and branched vascular architectures. Skylar-Scott et al. created a perfusable cardiac tissue that demonstrated synchronous beating over seven days.

#### 3.6.5 Direct 3D bioprinting

In contrast to its indirect counterpart, this technique offers a significant advantage by enabling the concurrent deposition of cellular components alongside the vascular architecture within a single, cohesive printing process. This approach streamlines the fabrication of tissue constructs and allows for precise spatial control over cell placement and vascular patterning. Such precision is critical for mimicking the complex, heterogeneous nature of native tissues. In tissue vascularization, a commonly used direct 3D bioprinting technique is extrusion-based bioprinting (EBB). As discussed previously, EBB involves the continuous deposition of bioinks containing cells and biomaterials through a nozzle via pneumatic pressure or mechanical (piston or screw) forces (246). It is ideal for fabricating multi-material constructs and those with varied compositions. The rheology of the bioink, which includes viscosity, shear thinning behavior, and yield stress, determines how the ink flows through the nozzle and behaves upon deposition (247). It is essential to use bioinks with appropriate rheological properties that support cellular activities and the formation of tubular structures.

#### 3.6.6 Coaxial bioprinting

Traditional EBB faces challenges in fabricating multilayered or simple tubular vasculatures. Coaxial bioprinting has emerged as a modality to address this limitation (248). It leverages the concentric extrusion of multiple biomaterials through coaxial nozzles, enabling the simultaneous deposition of core (bioink) and shell (crosslinker) materials (249). Natural biomaterials, such as alginate, collagen, GelMA, and chitosan, are frequently used as coaxial bioinks and have superior biocompatibility relative to synthetic materials (250). Coaxial bioprinting enables the fabrication of intricate constructs, solid or hollow tubes, in a single step. The specific configuration of the bioink and crosslinker within the coaxial nozzles dictates the resultant construct type. For instance, in creating hollow tubes that mimic tubular vascular structures, the crosslinker is extruded from the inner nozzle, while the bioink is dispensed from the outer nozzle (251). Several parameters affect the bioprinting outcome, including nozzle diameter, bioink viscosity, and printing extrusion rate (250). Therefore, these factors must be carefully considered to achieve optimal results.

#### 3.6.7 Freeform reversible embedding of suspended hydrogels (FRESH) bioprinting

**Figure 7:**
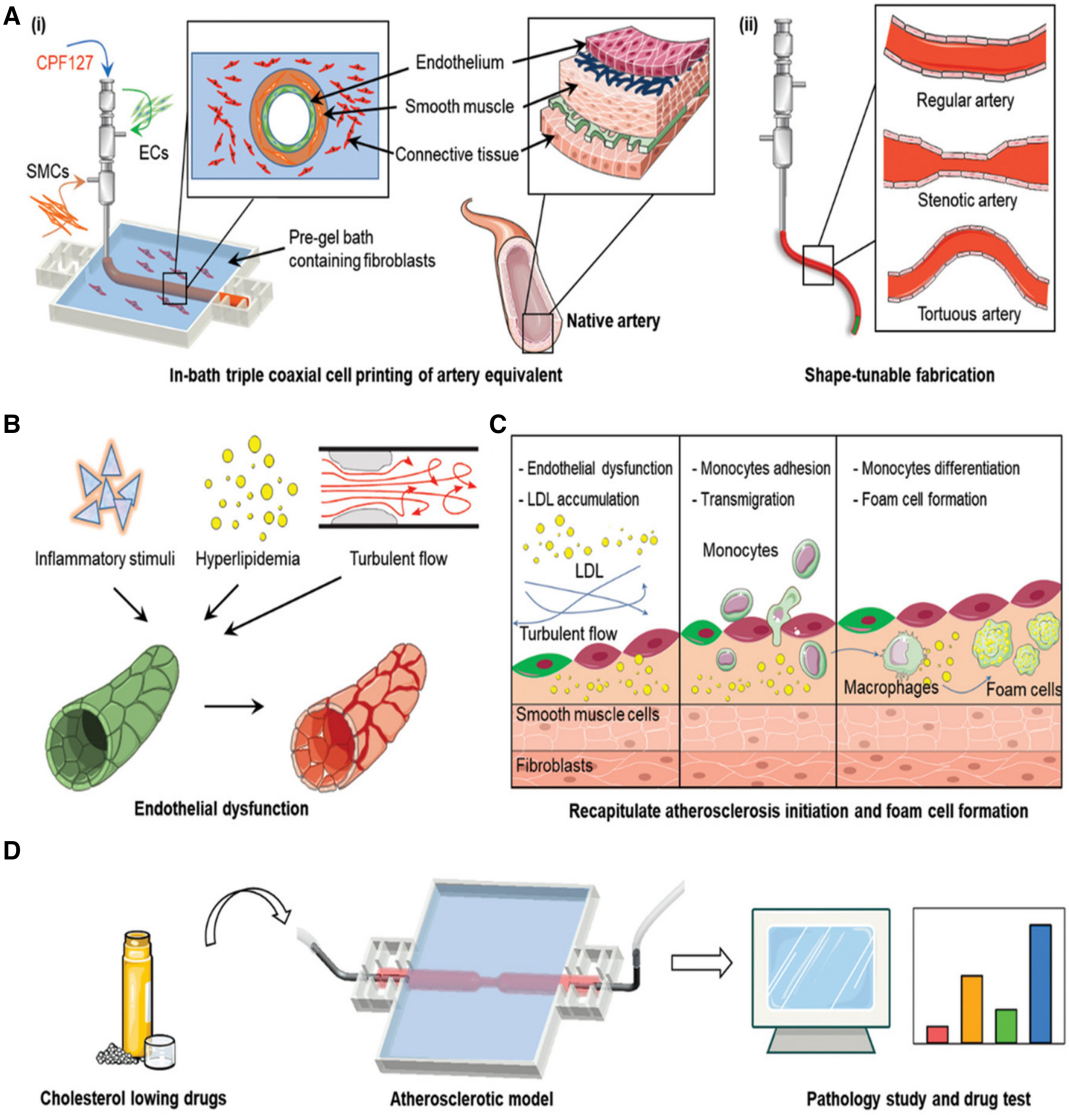
3D bioprinting to model atherosclerosis. (**A**) (**i**) The process of engineering an artificial artery using a bioprinting technique known as in-bath triple coaxial cell printing. Endothelial cells (ECs), smooth muscle cells (SMCs), and pre-gel solution containing fibroblasts are co-extruded to form concentric layers that mimic the structure of a native artery. (**ii**) Comparing the structure of a regular artery with that of a stenotic artery, highlighting the narrowed lumen due to plaque buildup, and a tortuous artery, which shows irregular winding. (**B**) The progression of atherosclerosis, starting with inflammatory stimuli and hyperlipidemia, leads to turbulent blood flow. This results in endothelial dysfunction, represented by the transition from a healthy endothelial-lined vessel to one with an irregular, disrupted lining. (**C**) A more detailed look at the cellular events during atherosclerosis development. It includes LDL oxidation beneath the endothelium, monocyte adhesion and transmigration, differentiation into macrophages, and foam cell formation—critical steps in plaque development. (**D**) The application of the atherosclerotic model for testing cholesterol-lowering drugs. It implies that the model can be used to observe the effects of these drugs on plaque formation, which is quantified in the bar graphs to the right, indicating a methodology to evaluate the pathology of atherosclerosis and drug efficacy. Used with permission from Gao et al. (256) © 2020 Wiley-VCH GmbH.

One of the limitations of coaxial bioprinting is the trade-off between the biomimetic quality of materials and their ease of fabrication. Since the printed materials are deposited on a flat surface, using mechanically weak bioinks increases the likelihood of loss of fidelity of the construct (252). A recent solution involves 3D bioprinting into suspension media as a supportive bath (253). This approach is known as FRESH (253, 254). Here, the bioink (containing cells and biomaterials) is extruded directly into a support bath, allowing the direct construction of complex, cell-laden vascular channels with high fidelity (253). The support bath is a thermoreversible hydrogel supporting the vascular structure during printing (255). After the printing process, the support bath is liquefied, leaving the intact, directly printed vascular structure. This approach can be combined with coaxial bioprinting to create complex tissues with high structural fidelity and biomimicry. Gao et al. constructed a sophisticated *in vitro* atherosclerosis model using a novel 3D in-bath coaxial cell printing technique to create a triple-layered artery equivalent with tunable geometries (Figure 7) (256). Vascular tissue-derived decellularized ECM (VdECM) was used as the bath material, demonstrating favorable rheological properties. This model enabled the study of atherosclerosis by co-culturing vascular cells under turbulent flow conditions. There has been a surge in studies utilizing FRESH bioprinting for tissue engineering. A comprehensive review can be found in Shiwarski et al. (254). ECM patterning approaches can serve as alternatives to 3D bioprinting, offering unique advantages and limitations. Next, we discuss vascular corrosion casting and viscous finger patterning.

#### 3.6.8 Vascular corrosion casting

**Figure 8:**
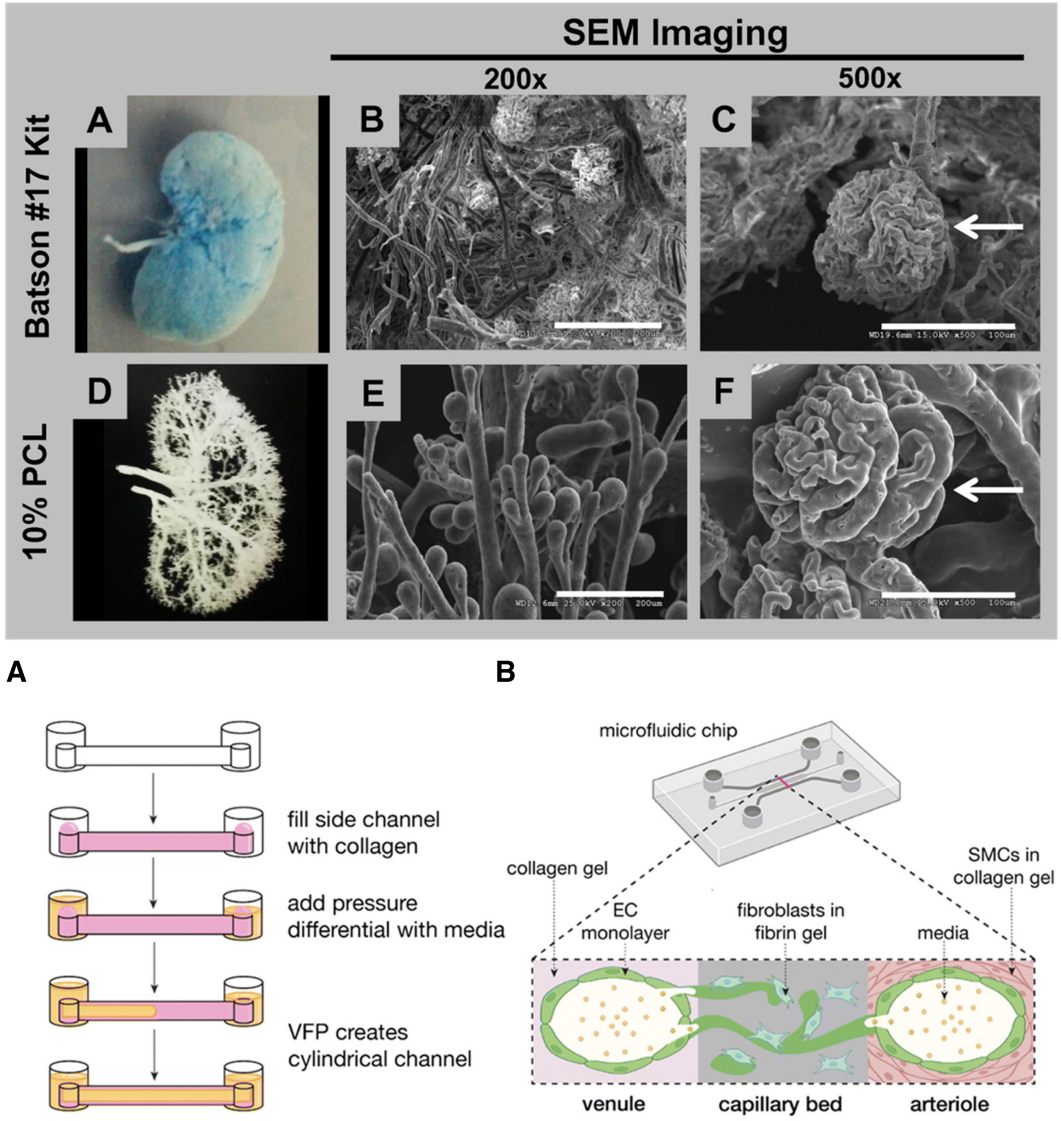
Vascular corrosion casting and viscous finger patterning techniques for creating vasculature. (**Top**): (**A**) The gross appearance of a vascular corrosion cast made using Batson #17 compound, a commonly used material for replicating fine vascular structures in the kidney. (**B**) and (**E**) SEM imaging at 200× magnification, revealing the network of vessels in the cortex region of the casts made from Batson #17 and PCL, respectively. (**C**) and (**F**) Further magnify the details of the casts at 500×, with a scale bar of 100 μm. The arrows in these images point to the glomerular capillaries preserved in the casts. (**D**) A similar gross view of a cast made from 10% polycaprolactone (PCL), a biodegradable polymer gaining traction in biomedical applications for its versatility and compatibility with body tissues*.* Used with permission from Huling et al. (257) Copyright © 2016 Acta Materialia Inc. Published by Elsevier Ltd. All rights reserved. (**Bottom**): (**A**) The viscous finger patterning process. Initially, a microfluidic channel is filled with collagen gel. Subsequent application of a pressure differential using media causes the collagen to deform and retract, creating a hollow, cylindrical channel within the gel. This channel serves as a mold for the vascular structures. (**B**) Cross-sectional view of the resulting microvascular networks (MVNs) within the microfluidic device. It shows an endothelial cell (EC) monolayer lining the inner walls of the channel, simulating the vessel lining. Surrounding the endothelial layer are smooth muscle cells (SMCs) in a separate collagen gel compartment, representing the muscular layer of arterioles. Adjacent to the SMCs, fibroblasts embedded in a fibrin gel depict the supportive connective tissue surrounding microvessels. The assembled structure is encased within a microfluidic chip, allowing for media flow and nutrient exchange. Used with permission from Chen et al. (258) licensed under CC BY-NC.

Huling et al. developed a cost-effective and straightforward method for creating biomimetic microvascular scaffolds using vascular corrosion casting tailored for pre-vascularizing engineered tissues (Figure 8; Top) (257). Utilizing polycaprolactone (PCL)-derived kidney vascular casts, the authors demonstrated the capability to replicate rat renal tissue architecture and form collagen-based scaffolds that can be perfused, endothelialized, and incorporated into hydrogel constructs. This biofabrication approach is an alternative to 3D bioprinting, offering a tissue-specific approach in tissue engineering. Nonetheless, the application of vascular casting faces certain limitations. These include the incomplete replication of native microvasculature in the vascular casts and challenges associated with processing these casts into scaffolds suitable for endothelialization. Some of these limitations may be specific to the use of PCL in vascular casting. In a subsequent investigation, the same research team evaluated the efficacy of these pre-vascularized scaffolds in kidney regeneration using a rat model (259). The implanted scaffolds facilitated both vascularization and the formation of renal tubules. Notably, these effects were potentiated when the scaffolds were combined with human renal cells, indicating a synergistic enhancement in renal tissue regeneration.

#### 3.6.9 Viscous finger patterning (VFP)

VFP, a phenomenon observed in fluid dynamics, occurs when a less viscous fluid infiltrates a more viscous one, creating patterns reminiscent of fingers or branches (260). Bischel et al. first used this approach to efficiently pattern lumens within type I collagen hydrogels in microchannels (261). The authors demonstrated its successful application in generating diverse channel geometries and multiple hydrogel layers. In tissue vascularization, VFP can be used to mimic the intricate branching structures of blood vessels. This involves injecting a bioengineered, less viscous material into a more viscous, cell-laden hydrogel matrix, forming a pattern (262). The key determinants of pattern complexity and size involve controlling the viscosity contrast between the two fluids and the injection pressure. The generated patterns are then seeded with endothelial cells to form perfusable vascular structures. Tsai et al. recently used VFP as a template to engineer vascular network-like structures (263). One of the advantages of VFP is its suitability for high-throughput applications because of its compatibility with automated liquid handling systems (261). However, scaling down to capillary size remains a limitation of this method. To address this limitation, different patterning methods can be combined to replicate the microvessel hierarchy. Recently, Chen et al. generated an interconnected arterial-capillary-venous system using viscous finger patterning and passive pumping for larger lumens and self-assembly for capillaries (Figure 8; Bottom). This fluidically interconnected system allowed for detailed studies of vessel permeability, vasoconstriction, and cell circulation behaviors like arrest and extravasation (258).

### 3.7 Incorporation of biophysical factors

Post-bioprinting, the nascent vessels mature within a carefully controlled biophysical environment tailored to facilitate their functional development. This maturation process is critically influenced by several biophysical factors, paramount among them is the mechanotransduction effects induced by shear stress. This stress, generated by blood flow within the vessels, is crucial in guiding the morphogenesis, differentiation, and alignment of endothelial cells (264). This mechanical stimulation is not just a passive response but an active driver in the cellular adaptation and vascular remodeling process. Traditional static culture systems are limited in replicating these dynamic mechanical environments, which are fundamental for mimicking the physiological function and stability of blood vessels. Integrating biophysical cues in the maturation process is thus essential for engineering vascularized tissues.

Bioreactors have provided controlled environments where vascularized tissues are subjected to defined mechanical forces, including shear stress, tension, and compression (265, 266). These systems have facilitated the study of cellular responses to mechanical cues in a more physiologically relevant environment. In addition, bioreactors permit the regulation of nutrient and oxygen gradients, thus supporting the generation of more complex organoid structures with enhanced cell viability (267). Perfusion bioreactors mimic the dynamic flow of blood, delivering mechanical stimulation and ensuring the continuous exchange of nutrients and waste products (268, 269). Sodian et al. developed a novel cell seeding and perfusion system for fabricating vascular tissues *in vitro*, incorporating biomechanical stimuli for vascular conditioning (270). Notably, the system allows dynamic cell seeding onto scaffolds and long-term tissue conditioning with adjustable flows and pressures.

In addition to shear stress, bioreactors can also provide cyclic strain (271), uniaxial tensile strain (272), and biaxial stretch (273). Huang et al. and Goodhart et al. engineered and validated bioreactors that apply biaxial and cyclic strain, respectively, to tissue-engineered vessels and spatially selected scaffolds (273, 274). Similarly, Subramanian et al. and Breen et al. designed bioreactors that administer uniaxial tensile strain and simultaneous wall shear stress and tensile forces, respectively, to cell-laden constructs and substrates (272, 275). Omid et al. used a bioreactor applying cyclic tensile and shear stresses to enhance cell integration and scaffold compactness in a decellularized ECM (276). Furthermore, Bono et al. introduced a dual-mode bioreactor for creating and stimulating collagen-based vascular constructs (271). This system enhanced the biomechanical properties of smooth muscle cell-laden constructs by applying cyclic strain, improving matrix compaction and cell distribution compared to static cultures. These bioreactors have successfully enhanced cell viability, growth, and matrix organization.

In addition to bioreactors, microfluidic devices play an essential role in the perfusion and maturation of vascularized tissues (24). Utilizing advanced microfabrication techniques, such as soft lithography and photolithography, these devices are designed with channel architectures that precisely control the fluidic environment (277, 278). The microfluidic channels facilitate the directed flow of cell culture media, often enriched with angiogenic factors to promote vascular development (279). Furthermore, critical to their functionality is the accuracy in channel size and geometry, which establishes physiological flow conditions and shear stress. These biophysical factors are instrumental in modulating endothelial cell morphology and function, directly influencing the formation and maturation of vascular structures. A notable application of the technology is in the development of organ-on-a-chip systems.

Organ-on-a-chip technology, advanced microfluidic cell culture, has emerged as a promising tool for drug development, precision medicine, and disease modeling for its ability to replicate human physiological conditions (280). This innovative system incorporates dynamic perfusion systems that emulate *in vivo* blood flow, essential for the maturation of intricate vascular structures. This platform manipulates shear stress and nutrient exchange, critical factors in endothelial cell differentiation and vessel functionality. The high-fidelity simulation of organ-specific microenvironments facilitates a deeper understanding of tissue responses to pharmacological agents. Within the last decade, several microfluidic on-chip devices have been created for the brain (281), heart (282), intestine (283), kidney (71), liver (284), lungs (285), ovary (286), pancreas (287), neurons (288), placenta (289), prostate (290), retina (291), and blood vessels (292). Given the ubiquity of its application across diverse tissue types, this technology represents a paradigm shift, enabling the creation of more physiologically relevant models that closely mirror human tissue dynamics, thereby enhancing the predictive accuracy of preclinical studies.

## 4 Conclusions and future outlook

The field of vascular biology remains central to our understanding of diverse physiological processes and pathologies. The vascular system, characterized by its intricate network of blood vessels, serves as a conduit for oxygen and nutrient delivery and functions in tissue regeneration, inflammation, and waste removal. Dysfunctions within this system underpin numerous diseases, such as atherosclerosis (293), diabetic retinopathy (294, 295), and cancer metastasis (296). Therefore, a reliable *in vitro* model of blood vessels is invaluable for insights into disease pathogenesis and therapeutic interventions. Moreover, tissue and organoid vascularization is pivotal in regenerative medicine, serving as the basis for fabricating functional organ substitutes. By replicating the intricate network of blood vessels, it addresses the critical issue of organ shortage. The landscape of vascular tissue and organoid engineering has undergone significant evolution, driven by the urgent need to alleviate the global crisis of organ scarcity and transform patient care by modeling a wide array of vascular diseases.

A vital aspect of this evolution is the progress in differentiating hPSCs into vascular cells. This advancement has provided a robust platform for disease modeling and drug screening. However, there are still challenges to current protocols. Achieving lineage-specific differentiation, maintaining cellular maturity, and replicating the complex vascular architecture, especially that of larger vessels, remain areas requiring further research.

3D bioprinting, particularly extrusion-based bioprinting, has emerged as a groundbreaking technology in tissue vascularization. Techniques such as SWIFT and FRESH have demonstrated remarkable success in fabricating thick, perfusable vascular networks. These approaches offer the potential to overcome traditional limitations of tissue depth and nutrient diffusion, presenting a promising avenue for creating more physiologically relevant constructs.

Despite these recent advances, current challenges with 3D bioprinting include achieving high-resolution vascular structures and ensuring the mechanical stability of the printed tissues. Developing and improving bioinks and smart hydrogels have been instrumental in this area. Innovations in bioink formulations have enhanced cellular compatibility and functionality. Smart hydrogels offer dynamic, stimuli-responsive environments conducive to vascular growth and maturation.

The integration of microfluidics in tissue engineering has been a significant step forward. The capacity for *in vitro* perfusion of vascularized tissues and organoids offers a more accurate mimicry of *in vivo* conditions, thereby enhancing the physiological relevance of these models. This approach circumvents the ethical and scientific complexities of implantation into animal hosts. Nonetheless, it remains a challenge to achieve full intraluminal *in vitro* perfusion without microfluidics (103).

The field of vascular tissue and organoid engineering stands at a promising juncture. The continued interdisciplinary collaboration between cell biology, materials science, and engineering is vital for overcoming the current challenges and unlocking the full potential of *in vitro* vascularized tissues and organoids. As we advance, the focus will likely shift towards enhancing the fidelity and functionality of engineered constructs, optimizing scalability, and ensuring reproducibility. The ultimate goal remains the development of reliable, patient-specific vascular models that can revolutionize personalized medicine and provide novel insights into the mechanisms of vascular diseases.
